# Scalable synthesis of Cu–Sb–S phases from reactive melts of metal xanthates and effect of cationic manipulation on structural and optical properties

**DOI:** 10.1038/s41598-020-80951-5

**Published:** 2021-01-21

**Authors:** Tahani Alqahtani, Malik Dilshad Khan, David J. Lewis, Xiang Li Zhong, Paul O’Brien

**Affiliations:** 1 School of Physics, Imam Abdulrahman Bin Faisal University, Dammam, Saudi Arabia; 2 Institute of Physical Chemistry, Polish Academy of Sciences, Kasprzaka 44/52, Warsaw, 01-224 Poland; 3 Department of Materials, The University of Manchester, Oxford Road, Manchester, M13 9PL UK; 4 Department of Chemistry, The University of Manchester, Oxford Road, Manchester, M13 9PL UK

**Keywords:** Chemistry, Materials science

## Abstract

We report a simple, economical and low temperature route for phase-pure synthesis of two distinct phases of Cu–Sb–S, chalcostibite (CuSbS_2_) and tetrahedrite (Cu_12_Sb_4_S_13_) nanostructures. Both compounds were prepared by the decomposition of a mixture of *bis*(*O*-ethylxanthato)copper(II) and *tris*(*O*-ethylxanthato)antimony(III), without the use of solvent or capping ligands. By tuning the molar ratio of copper and antimony xanthates, single-phases of either chalcostibite or tetrahedrite were obtained. The tetrahedrite phase exists in a cubic structure, where the Cu and Sb atoms are present in different coordination environments, and tuning of band gap  energy was investigated by the incorporation of multivalent cationic dopants, i.e. by the formation of Zn-doped tetrahedrites Cu_12−x_Zn_x_Sb_4_S_13_ (x = 0.25, 0.5, 0.75, 1, 1.2 and 1.5) and the Bi-doped tetrahedrites Cu_12_Sb_4−x_Bi_x_S_13_ (x = 0.08, 0.15, 0.25, 0.32, 0.4 and 0.5). Powder  X-ray diffraction (p-XRD) confirms single-phase of cubic tetrahedrite structures for both of the doped series. The only exception was for Cu_12_Sb_4−x_Bi_x_S_13_ with x = 0.5, which showed a secondary phase, implying that this value is above the solubility limit of Bi in Cu_12_Sb_4_S_13_ (12%). A linear increase in the lattice parameter *a* in both Zn- and Bi-doped tetrahedrite samples was observed with increasing dopant concentration. The estimated elemental compositions from EDX data are in line with the stoichiometric ratio expected for the compounds formed. The morphologies of samples were investigated using SEM and TEM, revealing the formation of smaller particle sizes upon  incorporation of  Zn. Incorporation of Zn or Bi into Cu_12_Sb_4_S_13_ led to an increase in band gap energy. The estimated band gap energies of Cu_12−x_Zn_x_Sb_4_S_13_ films ranges from 1.49 to 1.6 eV, while the band gaps of Cu_12_Sb_4−x_Bi_x_S_13_ films increases from 1.49 to 1.72 eV with increasing x.

## Introduction

Copper-based materials have attracted considerable attention due to their potential use as absorber materials for harvesting solar energy and as thermoelectric materials. Among such materials, copper indium gallium selenide (CIGS) has been considered as an excellent absorber material for solar cells with efficiency as high as 20.8%^1,2^. However, this material is associated with high cost and scarce elements (In and Ga). Cu_2_ZnSnS_4_ (CZTS) is a promising alternate earth abundant and low-toxic absorber material with a direct band gap and strong light absorption coefficient of over ∼10^4^ cm^−1^^3,4^. Nevertheless, because of its complex phase diagram, obtaining CZTS as a single phase is a significant challenge^5^.

Recently, ternary Cu–Sb–S systems have  shown considerable potential as alternate absorber materials for inexpensive photovoltaic energy generation. These materials have close to optimal band gap energies for absorption of solar photonic flux combined with, high absorption coefficients and consist of modestly earth-abundant, and relatively non-toxic elements^6–9^. The Cu–Sb–S system exists in four crystallographic phases, i.e. chalcostibite (CuSbS_2_), tetrahedrite (C_12_Sb_4_S_13_), skinnerite (Cu_3_SbS_3_) and fematinite (Cu_3_SbS_4_). The band gap energies of these phases, vary between 1.1 and 1.8 eV and they show excellent absorption coefficients (i.e. over 10^5^ cm^−1^) with p-type electrical conductivity^6,9–15^. Among these four phases, chalcostibite CuSbS_2_ (*E*_g_ ~ 1.4–1.5 eV)^9,11^ and tetrahedrite Cu_12_Sb_4_S_13_ (*E*_g_ ~ 1.2–1.7 eV)^13,16^ have promising band gap values required for maximum solar energy utilization^6^.

Chalcostibite CuSbS_2_ has been regarded as a substitute material to CuInS_2_ due to their analogous optical properties, with an added advantage of earth abundance of antimony and its lower cost compared to indium^17,18^. It has a direct band gap of 1.4–1.5 eV that is close to the optimum band gap range for solar energy conversion^19,20^, large absorption coefficient of 10^4^–10^5^ cm^−1^ and suitable electrical properties for solar cell applications^7,21,22^.

Another important phase of Cu–Sb–S, tetrahedrite Cu_12_Sb_4_S_13_, has recently attracted attention, as it has emerged as a potential material for energy conversion applications. With its naturally low lattice thermal conductivity and its anisotropic crystal structure, Cu_12_Sb_4_S_13_ is a candidate for thermoelectric energy generation via the Seebeck effect^23,24^. In addition, the strong absorption over a wide spectral range makes it useful as solar absorber material^8,25^. Naturally occurring tetrahedrite exists with varying compositions as (Cu, Ag)_10_(Cu, Zn, Fe, Cd, Hg)_2_(Sb, Te, Bi, As)_4_(S, Se)_13_ and can accommodate a range of substituents into the copper and antimony sites. The tetrahedrite has mixed Cu(I) and Cu(II) ions, which can be represented by (Cu^+^)_10_(Cu^2+^)_2_Sb_4_S_13_^6^. The presence of metal cations with + 1, + 2 and + 3 oxidation states, provides flexibility with respect to incorporation of various isovalent dopants. However, in cubic tetrahedrite copper and antimony ions have different coordination environments, and therefore it is difficult to ascertain which cationic dopant will significantly influence the electronic properties of the tetrahedrite. Partial substitution (doping) of the copper, antimony and chalcogen sites Cu_12−x_A_x_Sb_4−y_B_y_S_13−z_Se_z_ (A = M^2+^; B = M^3+^) by other elements is important to optimize the thermoelectric properties. These dopants could also lead to improved optical and magnetic properties. Pure (undoped) tetrahedrite Cu_12_Sb_4_S_13_ is generally prepared by solid-state reactions, which require high temperatures and long melting and annealing procedures that takes as long as three weeks to ensure that products are phase pure^24,26,27^. Solution-phase and wet chemical approaches to develop faster and simpler methods of preparing phase-pure tetrahedrite have been reported to address this^6,15,16,28–33^, and include hot injection^15^, solvothermal^16,33^, and spin coating or drop casting methodologies^31,34^. These approaches usually produce the tetrahedrite in the form of nanoscale particles and thin films, which show significantly different properties from those of bulk form. Rath and Haque et al.^34^ deposited chalcostibite or tetrahedrite thin films using mixture of alkyl xanthate complexes of Cu and Sb, by varying the stoichiometric ratios of the precursors. The mixture solution was spin coated or drop casted on the substrates, which were annealed to obtain thin films. A layer thickness of 50–100 nm was achieved by spin coating and 1–2 µm by drop casting. Weller et al.^35^ reported the solution-phase synthesis of tetrahedrite using a polyol process, which produced a nanostructured product (50–200 nm)^35^. It was shown that for these materials the thermopower was enhanced and thermal conductivity was decreased as compared to bulk tetrahedrite. Chen et al*.*^32^ prepared spherical tetrahedrite nanocrystals with controllable size by the colloidal chemical route. The band gap energy was tuned from 2.45 to 1.82 eV by changing the particle size from 2 to 16 nm. However, the presence of capping agents or sulfonating agents in these solution-phase approaches can negatively affect the thermoelectric properties^36^. In addition, these solution-phase methods require expensive solvents for both synthesis and subsequent purification, which can significantly limit their large scale production^37^.

This study reports a facile solvent-less method for the synthesis of orthorhombic chalcostibite and cubic tetrahedrite phases of copper antimony sulfide by using metal xanthate precursors. The absence of a solvent makes it environmentally friendly and economical as compared to hot injection and other colloidal routes^38^. The use of metal xanthates is beneficial as sulfur is already bonded to the metal atom in the precursor, which upon decomposition yields the metal sulfide, therefore eliminating the need of using an external sulfur source such as hydrogen sulfide. Likewise, the xanthate complexes are advantageous as they decompose at low temperatures (< 200 °C) and the by-products are volatile, which allows formation of clean and pure Cu–Sb–S materials at relatively low temperatures compared to solid state routes^39,40^. Such low temperatures are compatible with processing of these semiconductors onto polymeric substrates for flexible electronics.

We have demonstrate that two distinct phases of Cu–Sb–S, chalcostibite (CuSbS_2_) and tetrahedrite (Cu_12_Sb_4_S_13_), can be obtained by tuning the molar ratio of Cu to Sb xanthates in the direct pyrolysis of the powdered mixture. Furthermore, the band gap of tetrahedrite was tuned by doping of Zn and Bi using Zn and Bi xanthates respectively. We investigate the structural, morphological, compositional and optical properties of the systems.

## Experimental

### Chemicals

Potassium ethyl xanthogenate (96%), antimony(III) chloride (SbCl_3_, ≥ 99.95%), copper(II) chloride dihydrate (CuCl_2_ 2H_2_O, 99.99%), bismuth(III) chloride (BiCl_3_, ≥ 98%), Zinc(II) nitrate hexahydrate (Zn(NO_3_)_2_ 6 H_2_O, ≥ 98%), methanol (99.8%), ethanol (95.0%), chloroform (CHCl_3_, ≥ 99%), Hexane (C_6_H_14_, ≥ 99%) were purchased from Sigma-Aldrich.

### Materials Characterisation

The synthesized materials were characterized by similar techniques as used previously^41,42^. Briefly, elemental analysis and thermogravimetric analysis were performed in the micro-analytical laboratory of the University of Manchester. Mettler Toledo TGA/DSC1 star model was utilised to obtain the TGA data, at a temperature between 30 and 600 °C and a heating rate of 10 °C min^−1^ under N_2_. X-Pert diffractometer was used to acquire p-XRD data, equipped with a Cu–K_α_ source (1.54059 Å). Each sample was scanned between 10° and 80° with 0.02° steps and 3 s per step, at 40 kV and 30 mA. Scanning electron microscopy (SEM) and energy-dispersive X-ray spectroscopy (EDX) measurements were obtained via a Philips XL30 FEG microscope, with an accelerating voltage of 10–20 kV. Raman spectra were recorded using a Renishaw 1000 Micro-Raman microscope with a 514 nm Argon ion laser. Transmission electron microscope (TEM) imaging and diffraction was performed using Tecnai F30 TEM operated at 300 kV.

### Synthesis of antimony(III) ethylxanthate, Sb[S_2_COEt]_3_

The precursor was synthesized by using similar method as reported previously^41,42^. Briefly, potassium ethylxanthate (8.0 g, 49.9 mmol) was dissolved in ethanol (150 mL), followed by a gradual addition of antimony(III) chloride (3.79 g, 16.6 mmol) solution in ethanol (50 mL), with constant stirring for one hour at room temperature. The reaction mixture was filtered, product was washed with ethanol and DI water, dried under vacuum and recrystallised from chloroform to give bright yellow solid, yield: 7.1 g, 88%; m.p. 90.3 °C. Elemental analysis: calc. for Sb[S_2_COEt]_3_: C, 22.25%; H, 3.11%; S, 39.55%; Sb, 25.08%. Found: C, 22.61%; H, 3.07%; S, 39.71%; Sb, 24.15%. IR (cm^−1^): 1220 ν(C–O); 1020 ν(C–S). ^1^H-NMR (CDCl_3_): δ 4.61 (q, J = 7.1 Hz, 2H, CH_2_), δ 1.44 (t, J = 7.1 Hz, 3H, CH_3_). ^13^C NMR: δ 207.02 (S_2_C), δ 72.07 (CH_2_), δ 13.9 (CH_3_).

### Synthesis of copper(II) ethylxanthate, Cu[S_2_COEt]_2_

Cu[S_2_COEt]_2_ was prepared following the same method outlined above for Sb[S_2_COEt]_3_, however, copper(II) chloride dihydrate (4.25 g, 24.9 mmol) was used as the copper source, The resulting dark yellow solid was washed with distilled water and methanol and dried in vacuum. Yield 5.7 g, 75%; m.p. 170 °C. Elemental analysis: calc. for Cu[S_2_COEt]_2_: C, 23.3%; H, 3.3%; S, 41.3%; Cu, 20.5%. Found: C, 22.9%; H, 3.2%; S, 40.9%; Cu, 20.8%; IR (cm^−1^): 1190 ν(C–O); 1007 ν(C–S).

### Synthesis of bismuth(III) ethylxanthate, Bi[S_2_COEt]_3_

Bi[S_2_COEt]_3_ was prepared by following the method outlined above for Sb[S_2_COEt]_3_^41,42^, however, bismuth(III) chloride (5.24 g, 16.6 mmol) was used as the bismuth source. The product was recrystallised from chloroform to give dark yellow solid. Yield 8.2 g, 86%; m.p. 110 °C. Elemental analysis: calc. for Bi[S_2_COEt]_3_: C, 18.86%; H, 2.64%; S, 33.53%; Bi, 36.49%. Found: C, 19.14%; H, 2.65%; S, 33.46%; Bi, 36.47%; IR (cm^−1^): 1206 ν(C–O); 1018 ν(C–S). ^1^H NMR (CDCl_3_): δ 4.69 (q, J = 7.1 Hz, 2H, CH_2_), δ 1.49 (t, J = 7.1 Hz, 3H, CH_3_). ^13^C NMR: δ 206.99 (S_2_C), δ 71.3 (CH_2_), δ 13.9 (CH_3_).

### Synthesis of Zinc(II) ethylxanthate, Zn[S_2_COEt]_2_

Zn[S_2_COEt]_2_ was prepared following the same method outlined above for Sb[S_2_COEt]_3_^41,42^, however, zinc(II) nitrate hexahydrate (7.42 g, 24.9 mmol) was used as the zinc source. Recrystallization from chloroform resulted in white solid, yield 6.2 g, 80%; m.p. 120 °C. Elemental analysis: calc. for Zn[S_2_COEt]_2_: C, 23.4%; H, 3.3%; S, 41.7%; Zn, 21.3%. Found: C, 23.6%; H, 3.2%; S, 41.6%; Zn, 21.1%; IR (cm^−1^): 1206 ν(C–O); 1022 ν(C–S). ^1^H NMR (CDCl_3_): δ 4.55 (q, J = 7.1 Hz, 2H, CH_2_), δ 1.46 (t, J = 7.1 Hz, 3H, CH_3_). ^13^C NMR: δ 207.7 (S_2_C), δ 77.2 (CH_2_), δ 13.9 (CH_3_).

### Synthesis of Cu–Sb–S nanomaterials by melt thermolysis

Stoichiometric mixtures of copper xanthate (Cu[S_2_COEt]_2_) and antimony xanthate (Sb[S_2_COEt]_3_) were used to prepare Cu**–**Sb**–**S phases using melt method. In a typical procedure, different molar ratios of Cu[S_2_COEt]_2_ and Sb[S_2_COEt]_3_ complexes were grounded using a pestle and mortar for half an hour to ensure uniform mixing of the complexes. The homogenous mixture was then loaded evenly into a ceramic boat inside a reaction tube for heating. Subsequently, the reaction tube was heated in the furnace up to the chosen temperature (250 °C), under flow of nitrogen. After 1 h, the heating was turned off and the sample was allowed to cool to room temperature prior to collecting for characterisations.

### Deposition of Cu–Sb–S thin films by doctor blade

Doctor blade technique is a simple, scalable and cost-effective way for the fabrication of uniform film on a variety of substrates. A blade is positioned over the substrate at an adjustable gap height from the substrate. A suitable amount of precursor solution (slurry) is placed in the front of the blade. The blade is then swiped linearly across the substrate, generating a uniform wet film layer^43,44^. The coating thickness is controlled by adjusting the gap between the blade and the substrate and the final wet layer should be roughly half of this gap from the substrate. However, many other factors can influence the thickness of the film including the precursor solution concentration, coating speed, surface tension of solution and the blade geometry^45,46^. In a typical procedure, mixture of Cu and Sb xanthate precursors was grounded using pestle and mortar for half an hour to ensure uniform mixing. Therefore, this is mixed with a few drops of hexane to produce a precursor slurry. The precursor slurry was dropped onto the glass substrate and swiped linearly by the doctor blade, followed by heating at 250 °C for one hour under nitrogen. The thickness of the dry film was found to be approximately 1.5 ± 0.1 μm as measured by SEM.

## Result and discussion

In our melt synthesis of chalcostibite (CuSbS_2_) and tetrahedrite (Cu_12_Sb_4_S_13_), we have explored the use of the Cu xanthate (Cu[S_2_COEt]_2_) and Sb xanthate (Sb[S_2_COEt]_3_) as the Cu, Sb and S sources for the desired ternary sulfide material. The compositions of the obtained materials were adjusted by varying the molar ratio of the copper and antimony xanthate precursors. In addition, zinc xanthate (Zn[S_2_COEt]_2_) and bismuth xanthate (Bi[S_2_COEt]_3_) were also used for the preparation of the Zn and Bi doped tetrahedrite, respectively. Thermogravimetric analysis (TGA) (Supporting Information Fig. S1) shows that the decomposition from metal xanthates to the corresponding metal sulfides occur cleanly in one single step for all precursors at 150–200 °C. This indicates that the ternary copper antimony sulfide and quaternary Zn/Bi doped tetrahedrite materials can be obtained by the melt thermolysis of Cu, Sb, Zn or Bi xanthates at mild temperatures for a short period of time. Moreover, the by-products of the decomposition are volatile, allowing the clean formation of the materials without impurities.

### Characterisation of Chalcostibite CuSbS_2_ nanomaterials

**Figure 1:**
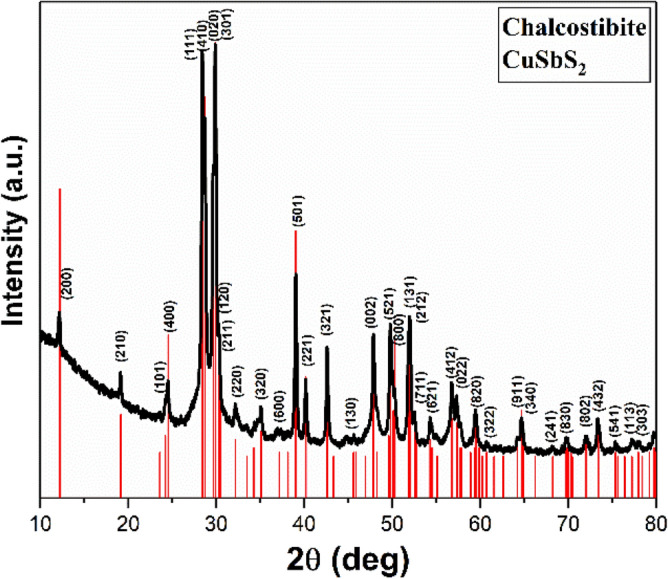
p-XRD pattern of chalcostibite CuSbS_2_ nanomaterials prepared using 1: 1 molar ratio of Cu and Sb xanthates at 250 °C. Red lines show the positions of Bragg reflections from the standard reference pattern of orthorhombic CuSbS_2_ (chalcostibite, ICDD: 01-088-0283).

The crystal structure and phase purity of the prepared cupper antimony sulfide nanomaterials are determined using powder-X-ray diffraction measurements (p-XRD). Figure 1 demonstrates the p-XRD patterns for the sample prepared using 1: 1 molar ratio of Cu and Sb xanthates at 250 °C. The position of the diffraction peaks in this powder pattern match well with a standard reference pattern for orthorhombic CuSbS_2_ (chalcostibite, ICDD: 00-044-1417).

**Figure 2:**
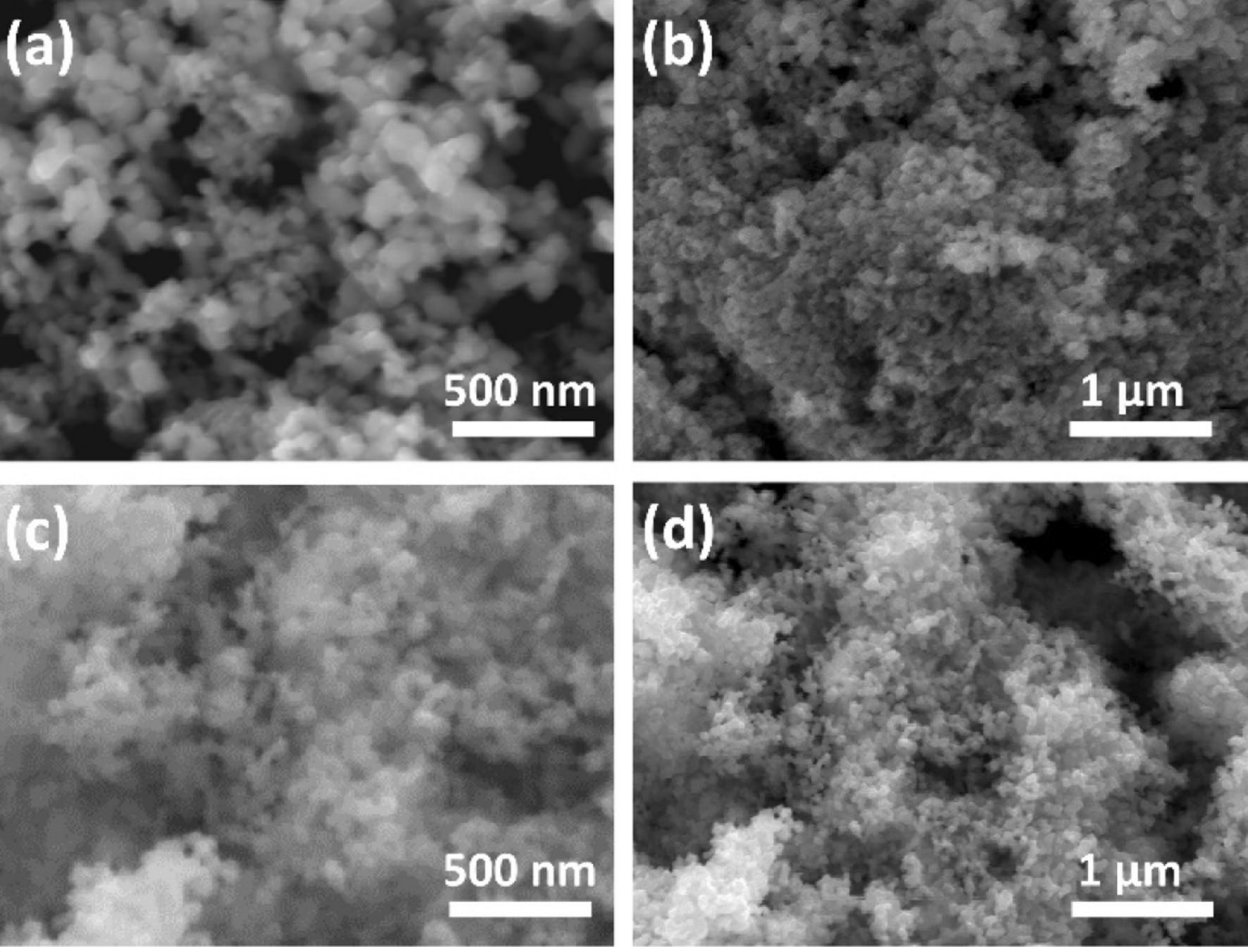
SEM images with different magnifications (**a**,**b**) for chalcostibite (CuSbS_2_) and (**c**,**d**) for tetrahedrite (Cu_12_Sb_4_S_13_).

Scanning electron microscopy (SEM) in combintion with energy dispersive X-ray (EDX) spectroscopy were used to analyse the surface morphology, particle size and chemical composition of the prepared Cu–Sb–S nanomaterials. SEM images of a chalcostibite CuSbS_2_ sample show spherical particles in nanometer-size range with an estimated average size of 89.8 ± 7.6, as presented in Fig. 2a,b. The small particle size is consistent with the Scherrer broadening observed in the p-XRD pattern presented earlier.

EDX spectra (Supporting Information Fig. S2a) display the characteristic emission lines of Cu, Sb and S. The chemical compositions of the chalcostibite CuSbS_2_ sample was obtained and compared to the expected values (Table 1). The Cu: Sb: S ratios are approximate with the expected compositions, but having slightly Cu-rich stoichiometry. EDX elemental mapping was used to determine the spatial distribution of elements in the synthesized CuSbS_2_ nanoparticles, as shown in Supporting Information Fig. S3a. The result reveals that the distribution of Cu, Sb and S elements is homogenous and uniform, with emission for all three elements co-localised in space as one may expect for such ternary phases.

**Table 1:** The content of Cu, Sb and S, in chalcostibite (CuSbS_2_) and tetrahedrite (Cu_12_Sb_4_S_13_) samples calculated from theoretical values and those found experimentally by EDX spectroscopy.

Sample	Cu (at%)	Sb (at%)	S (at%)	Stoichiometry
**CuSbS**_**2**_				
Expected	25	25	50	CuSbS_2_
Sample	30.1	21.2	48.8	Cu_1.2_Sb_0.85_S_1.95_
**Cu**_**12**_**Sb**_**4**_**S**_**13**_				
Expected	41.4	13.8	44.8	Cu_12_Sb_4_S_13_
3: 1 ratio	49.2	9.4	41.4	Cu_14.27_Sb_2.73_S_12_
2: 1 ratio	41.1	14.2	44.6	Cu_11.92_Sb_4.12_S_12.93_

**Figure 3:**
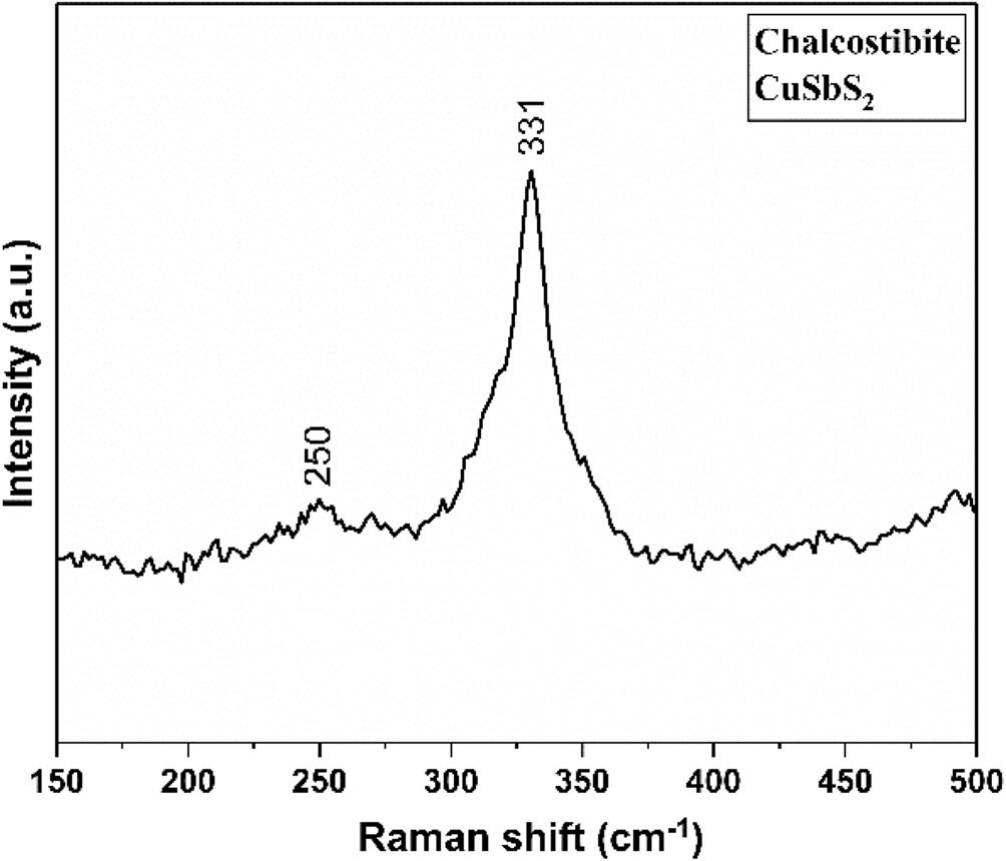
Raman spectrum of chalcostibite CuSbS_2_ prepared using a 1: 1 ratio of Cu and Sb xanthate powders at 250 °C.

Figure 3 shows the Raman spectrum (excitation wavelength of 514 nm) for the Chalcostibite CuSbS_2_. We observe a peak in the spectrum at 331 cm^−1^ and a peak of lesser intensity at 250 cm^−1^. Both peaks are associated with orthorhombic CuSbS_2_ Chalcostibite; the peak at 331 cm^−1^ is attributed to the vibration of Sb–S bonds, whilst the peak at 250 cm^−1^ is assigned to the vibration of Cu–S bonds in reference spectra^47–49^.

### Characterisation of Tetrahedrite Cu_12_Sb_4_S_13_ nanomaterials

**Figure 4:**
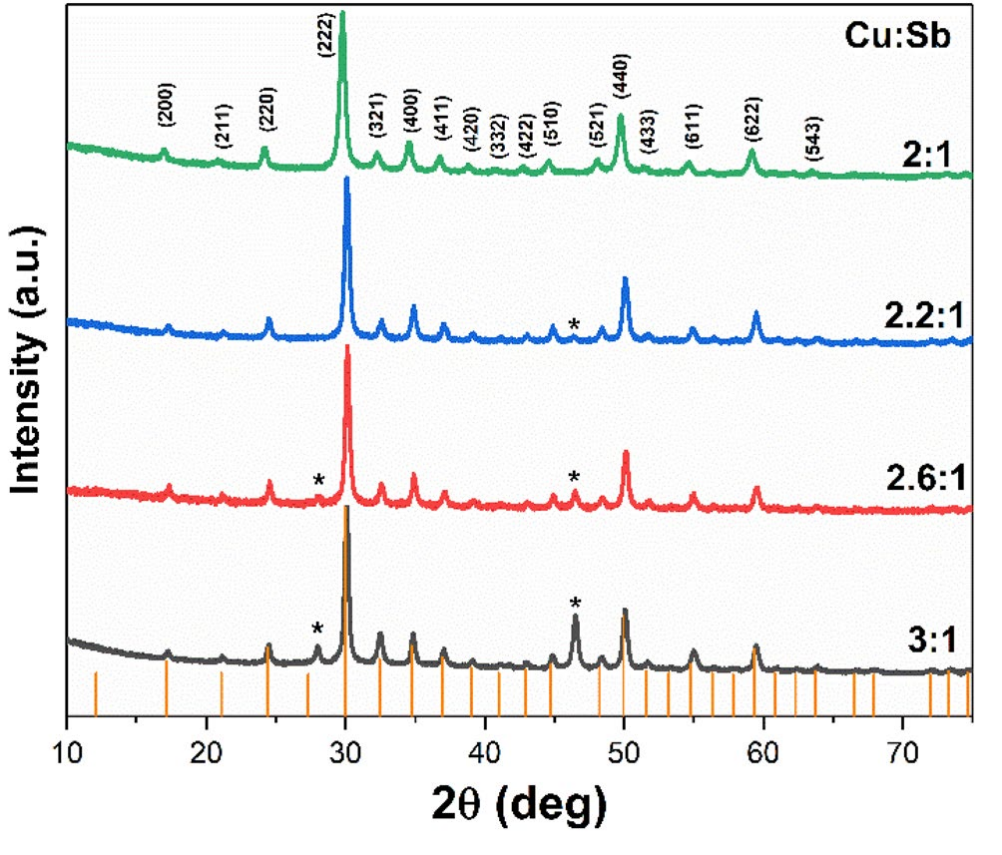
p-XRD patterns of tetrahedrite Cu_12_Sb_4_S_13_ nanomaterials obtained at different molar ratios of copper and antimony xanthate precursors. Orange lines are from the standard reference pattern for cubic Cu_12_Sb_4_S_13_ (tetrahedrite, ICDD: 01-088-0283). *Indicates the impurity peaks associated with formation of Cu_2−x_S (ICDD: 00-002-129).

Figure 4 presents the p-XRD patterns of tetrahedrite (Cu_12_Sb_4_S_13_) nanomaterials obtained with different molar ratios of copper and antimony xanthate precursors. Initially, the tetrahedrite was prepared using 0.3 mmol of Cu-xanthate and 0.1 mmol of Sb-xanthate at 250 °C for 1 h (i.e. molar ratio = 3:1). However, this procedure yielded tetrahedrite with extra impurity peaks observed around 27.9° and 46.5° corresponding to Cu_2−x_S ICDD: 00-002-1292 (and marked by * in Fig. 4). Accordingly, the tetrahedrite nanomaterials with various Cu/Sb ratios which were synthesised at different temperatures and times were investigated to find out the optimal conditions for formation of pure-phase tetrahedrite. Varying annealing time and temperature, did not remove the impurity peaks associated with Cu_2−x_S. However, we found that by decreasing the molar ratios of the copper/antimony xanthate precursors to 2.8:1, that the intensity of the reflections of the impurity peaks decreased. This effect was found to be monotonic upon further decrease in Cu:Sb molar ratio to 2.2:1, which further decreased the copper sulfide impurity. Ulitmately, by using 0.2 mmol of Cu-xanthate and 0.1 mmol of Sb-xanthate (i.e. molar ratio = 2:1) we found that we successfully produced a single phase corresponding to cubic tetrahedrite (Cu_12_Sb_4_S_13_ ICDD: 01-088-0283) without presence of any other phases in the X-ray powder pattern, as illustrated in Fig. 4. Therefore, we concluded that using 2:1 molar ratio of Cu and Sb xanthate precursors is optimal in obtaining phase-pure tetrahedrite. Many studies have revealed that the presence of a small amount of impurity phases in the synthetic tetrahedrite cannot be avoided^50–59^. Previously, phase pure tetrahedrite nanoparticles have been prepared either by using capping agents, such as thiols, or incorporation of different dopants^52,60,61^. However, this study indicates that the solventless pyrolysis of the metal xanthate is advantageous in that it has demonstrated successful formation of the single-phase tetrahedrite Cu_12_Sb_4_S_13_ of high purity in a short amount of time without the addition of any dopants or capping agents. The lattice parameter *a* of the Cu_12_Sb_4_S_13_ was calculated from the p-XRD data and found to be 10.368 Å, which is consistent with previosuly reported literature values for cubic tetrahedrite (ICDD: 01-088-0283).

Figure 2c,d show SEM images at low and high magnifications of pure tetrahedrite Cu_12_Sb_4_S_13_ prepared from 2:1 molar ratio of Cu and Sb xanthates. These images indicate the formation of pseudo-spherical particles at the nano-scale with an average size of 61 ± 8 nm. Similar morphologies for tetrahedrite nanoparticles (in the range of 50–200 nm) synthesised by modified polyol process have been reported^35^. The EDX spectra for tetrahedrite (Supporting Information Fig. S2b) displays the characteristic peaks of the three elements Cu, Sb and S, and the chemical compositions were obtained and compared to the expected values. For the tetrahedrite Cu_12_Sb_4_S_13_, EDX spectroscopy was performed on samples synthesized using different molar ratios of Cu and Sb xanthates (Table 1). The expected values of tetrahedrite Cu_12_Sb_4_S_13_ should have atomic % of Cu 41.4%, Sb 13.8% and S 44.8%. However, in the 3: 1 ratio we have obtained 49.2% of Cu, 9.4% of Sb and 41.4% of S, which is Cu-rich and Sb-deficient. In contrast, the final product of tetrahedrite using 2: 1 molar ratio of Cu and Sb is very close to the expected values for the pure tetrahedrite Cu_12_Sb_4_S_13_ (41.1 of Cu, 14.2 of Sb and 44.6 of S), giving the formula of Cu_11.9_Sb_4.1_S_12.9_. These results are consistent with the p-XRD results as the 3:1 ratio yielded an extra impurity phase, while using 2: 1 ratio exhibited a single tetrahedrite phase. Further inspection of TGA data for all complexes show that, beside antimony, the complexes decompose in a single step. The TGA of the antimony complex reveals that a major weight loss step occurs around 150 °C, and unlike other the complexes, undergoes another small weight loss step slightly below 250 °C. This may be attributed to an escape or volatilization of the residual product, which may slightly alter/decrease the amount of antimony content in tetrahedrite phase when 3:1 ratio is used, thereby resulting in copper rich product or introduction of impurity phase. Therefore a gradual decrease in the copper content result in the diminishing of the impurity phase and at 2:1 ratio, phase pure tetrahedrite was obtained. Both p-XRD and EDX spectroscopy revealed that the molar ratios of the Cu and Sb xanthate precursors plays an important role in the formation of single phase tetrahedrite Cu_12_Sb_4_S_13_. EDX elemental mapping was performed to determine the elemental distribution of the synthesized Cu_12_Sb_4_S_13_ nanoparticles, as shown in Supporting Information Fig. S3b. The result reveals that the distribution of Cu, Sb and S elements is homogenous and uniform. Furthermore, the decomposition of mixtures of Cu and Sb complexes in 3:1 and 2:1 ratio respectively, was also studied by TGA. It was observed that the complexes decompose completely at 180 °C, resulting in the formation of tetrahedrite phase (Supporting Information Fig. S4). The tetrahedrite phase was stable up to 400 °C, after which a marginally small weight loss was observed between 400 and 500 °C. After 500 °C, a significant change was noticed which may show degradation of tetrahedrite phase via sulfur loss or other volatile by-products.

Transmission electron microscopy (TEM) and selected area electron diffraction (SAED) were performed and presented in Fig. 9. TEM images of the tetrahedrite Cu_12_Sb_4_S_13_ nanoparticles show that the formation of agglomerated particles is in the nanometer range, with an estimated average size of 62 ± 9 nm, which is consistent with SEM results. Lattice fringes (Fig. 9b) were observed with estimated d-spacing of 2.99 Å, corresponding to the (222) lattice plane of cubic tetrahedrite Cu_12_Sb_4_S_13_^6^. The inset of Fig. 9b shows the SAED pattern of the tetrahedrite Cu_12_Sb_4_S_13_ nanomaterials, where circular rings were observed indicating the polycrystalline nature of the sample. There are six diffractions rings which correspond to the (220), (222), (400), (440), (611) and (622) lattice planes of the cubic tetrahedrite Cu_12_Sb_4_S_13_^62^, which is consistent with the p-XRD patterns of these materials (Fig. 4).

**Figure 5:**
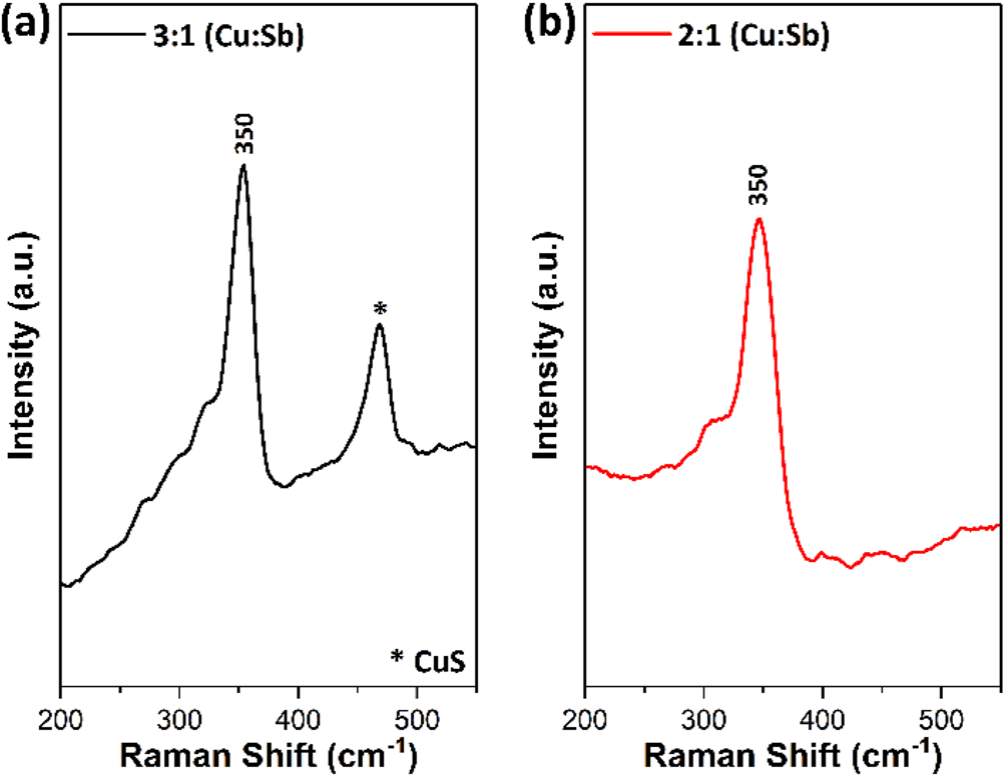
Raman spectra of tetrahedrite Cu_12_Sb_4_S_13_ samples obtained at (**a**) 3:1 and (**b**) 2:1 molar ratio of Cu and Sb xanthate precursors.

Raman spectra of tetrahedrite Cu_12_Sb_4_S_13_ samples obtained for the materials produced with different molar ratios of Cu and Sb xanthate precursors are displayed in Fig. 5. Two Raman modes were observed for the sample obtained using 3:1 ratio (Cu: Sb). The Raman peak observed at 350 cm^−1^ is attributed to the cubic tetrahedrite Cu_12_Sb_4_S_13_^63^. However, the Raman peak at 470 cm^−1^ corresponds to the binary Cu_2−x_S impurity phase^64,65^. For the 2: 1 ratio sample, only one vibrational mode was observed at 351 cm^−1^, which can be assigned to the symmetric stretching modes of the cubic tetrahedrite^66^. In this sample, no Raman peak is found for Cu_2−x_S (470 cm^−1^) impurity phase. These results correlate well with the p-XRD analysis, where extra impurity peaks were observed only for the 3:1 ratio sample. Hence, the Raman study indicates that the 2:1 ratio of Cu and Sb xanthate is the optimal ratio to obtain phase pure tetrahedrite.

### Characterisation of Zn^2+^ doped Cu_12_Sb_4_S_13_ nanomaterials

**Figure 6:**
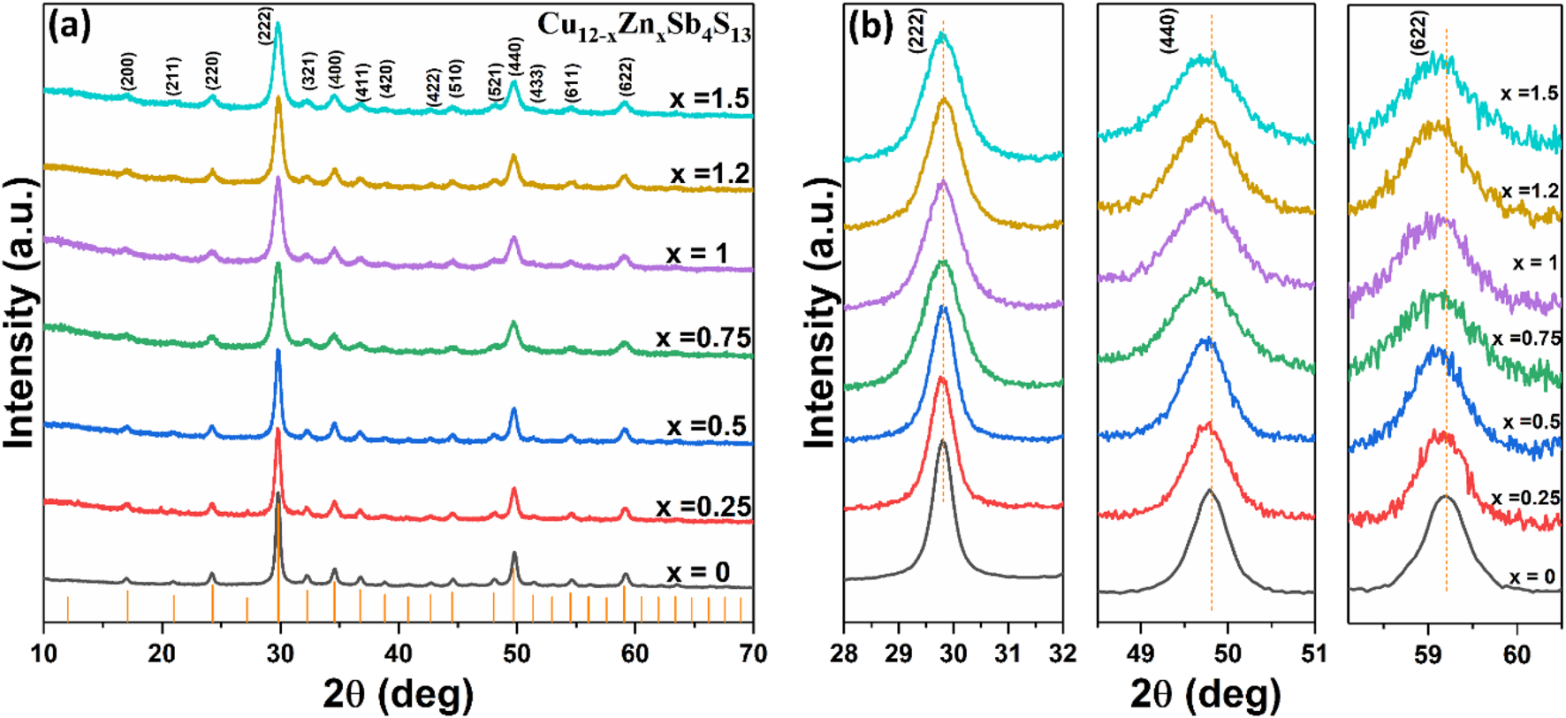
(**a**) p-XRD patterns of Cu_12−x_Zn_x_Sb_4_S_13_ (x = 0.25, 0.5, 0.75, 1, 1.2, 1.5) and (**b**) the three main diffraction peaks (222), (440) and (622) of Zn-doped tetrahedrite samples. Orange lines are from standard ICDD: 01-088-0283 for cubic Cu_12_Sb_4_S_13_ (tetrahedrite).

**Figure 7:**
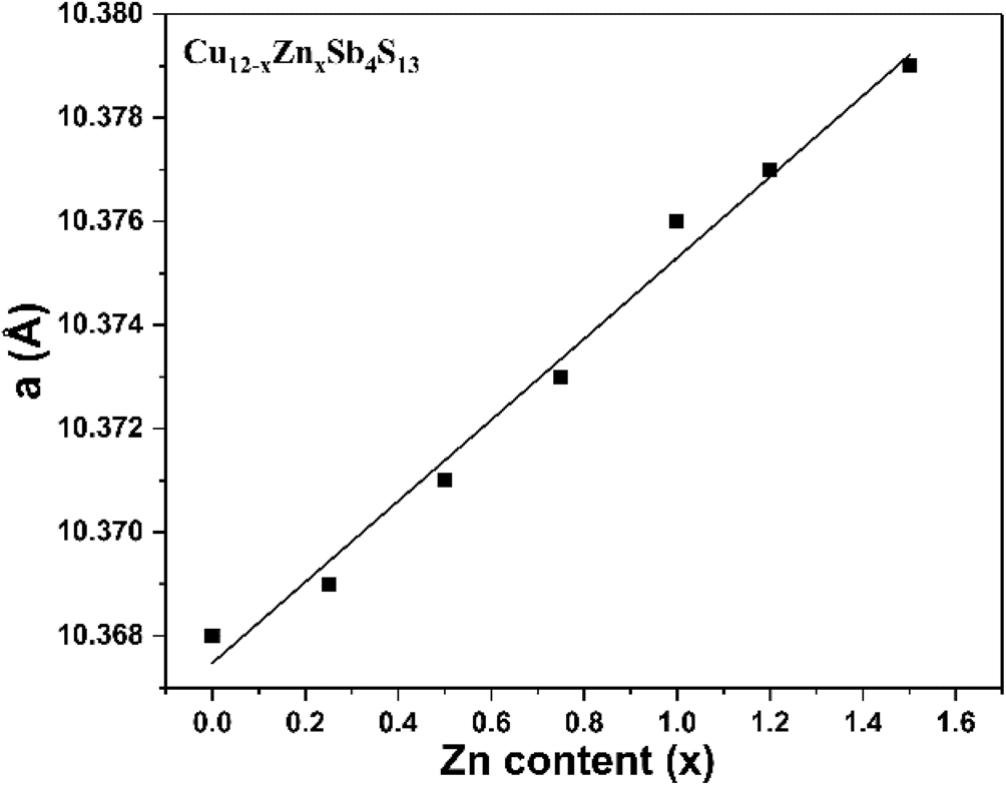
Lattice parameter *a* as a function of Zn stoichiometry (x) in Cu_12−x_Zn_x_Sb_4_S_13_ (x  =  0.25, 0.5, 0.75, 1, 1.2, 1.5).

Zn doped tetrahedrite nanomaterials were prepared by using the optimized quantities of Cu and Sb xanthate precursors (i.e. 2:1 ratio). p-XRD patterns (Fig. 6) reveal that the Zn-doped tetrahedrite Cu_12−x_Zn_x_Sb_4_S_13_ (x = 0.25, 0.5, 0.75, 1, 1.2 and 1.5) nanocrystals have the same cubic crystal structure as the undoped tetrahedrite nanoparticles (Cu_12_Sb_4_S_13_ ICDD: 01-088-0283). It has been widely reported that the substituted Zn atom replaces the Cu(1) site in CuS_4_ tetrahedra within the tetrahedrite structure^60,67–70^. As can be seen from the p-XRD, the Zn is incorporated into the tetrahedrite, as there is no change in crystal structure and there is no evidence of the formation of a secondary zinc-rich phase e.g. ZnSs. Figure 6b displays the three main diffraction peaks of Cu_12−x_Zn_x_Sb_4_S_13_ samples with various dopant levels, which are assigned to the (222), (440) and (622) Bragg planes of the tetrahedrite structure. The peaks were broadened as the Zn dopant level increased in the samples; this broadening effect is likely brought about by the changes in size of the nanocrystals, which we discuss later. Furthermore, the maximum intensity of the diffraction peak (222) showed no shift upon increasing the Zn doping content. However, the other two diffraction peaks, namely the (444) and (622) planes, displayed a small shift towards lower angle as the Zn dopant concentration increased in the samples. The shift of the diffraction peaks indicates an expansion in the lattice cell due to the atomic radius of Zn^2+^ (0.68 Å) which is slightly larger than that of Cu^1+^ (0.60 Å)^71^. Table 2 shows the unit cell lattice parameters *a* for Cu_12−x_Zn_x_Sb_4_S_13_ samples, which were calculated from the X-ray-diffraction data using the lattice relation for cubic structure; $d=\frac{a}{\sqrt{{h}^{2}+{k}^{2}+{l}^{2}}}$, where d is lattice plane spacing and (hkl) are the Miller indices of the plane^72^. The calculated lattice parameter *a* of all samples is plotted as a function of Zn composition, as illustrated in Fig. 7. The lattice parameter *a* was found to increase from 10.368 to 10.379 Å when increasing the Zn content in the samples from x = 0 to x = 1.5. Similar behaviour was observed in the Zn-doped tetrahedrite series by May et al.^73^ where the lattice parameter increases from 10.3221 Å for pure tetrahedrite to 10.3805 Å for Cu_10_Zn_2_Sb_4_S_13_.

**Table 2:** Lattice parameter *a*, EDX compositional analysis and the estimated optical band gap (*E*_g_) of Cu_12−x_Zn_x_Sb_4_S_13_ (x = 0.25, 0.5, 0.75, 1, 1.2, 1.5).

x	Composition	Lattice parameters *a* (Aͦ)	Elemental composition by EDX spectroscopy (atomic%)				Optical band gap energy, *E*_g_ (eV)
			Cu	Zn	Sb	S	
0	Cu_12_Sb_4_S_13_	10.368	41.1	0	14.2	44.6	1.49
0.25	Cu_11.76_Zn_0.24_Sb_4_S_13_	10.369	39.2	0.7	14.5	45.6	1.50
0.5	Cu_11.52_Zn_0.48_Sb_4_S_13_	10.371	38.2	1.7	15.1	44.9	1.50
0.75	Cu_11.28_Zn_0.72_Sb_4_S_13_	10.373	38.1	2.2	15.5	44.1	1.51
1	Cu_11.04_Zn_0.96_Sb_4_S_13_	10.376	37.8	2.6	14.8	44.7	1.52
1.2	Cu_10.8_Zn_1.2_Sb_4_S_13_	10.377	37.0	3.0	15.4	44.6	1.54
1.5	Cu_10.56_Zn_1.44_Sb_4_S_13_	10.379	35.9	4.4	15.6	44.2	1.60

**Figure 8:**
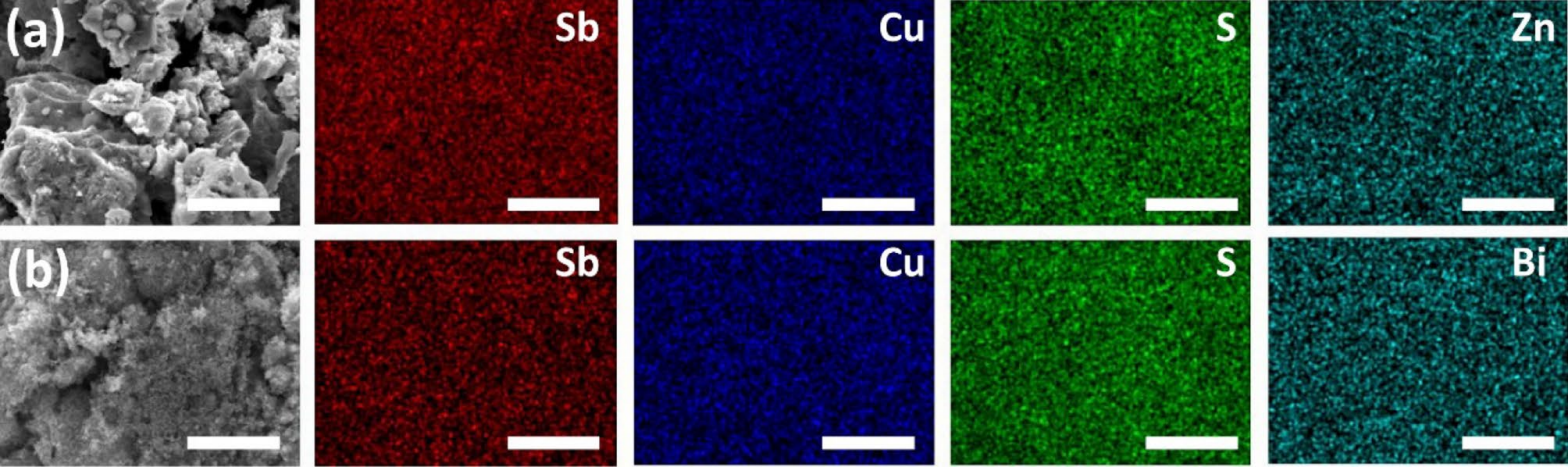
EDX elemental mapping (20 kV) of Cu Kα, Sb Lα, S Kα and Zn Kα/Bi Mα for (**a**) Cu_12−x_Zn_x_Sb_4_S_13_ (x = 1.5) and (**b**) Cu_12_Sb_4−x_Bi_x_S_13_ (x = 0.5). Scale bars = 5 µm.

SEM images that show the morphologies of the Zn-doped tetrahedrite Cu_12−x_Zn_x_Sb_4_S_13_ samples with different Zn contents are illustrated in Fig. S5 (Supporting Information). The SEM images reveal particles in the nano-size range with spherical to irregular shapes. Furthermore, the incorporation of the Zn into the tetrahedrites reduces the particle size from 61 ± 8 for pure tetrahedrite to 28 ± 3 nm for 12% Zn-doped tetrahedrite. This finding correlates well with p-XRD data collected for these samples as the peaks broaden with increasing Zn in the samples. A similar observation was reported by Battiston et al.^74^, who identified that adding Zn yielded a smaller particle size. EDX spectrscopy of the Cu_12−x_Zn_x_Sb_4_S_13_ (x = 0.25, 0.5, 0.75, 1, 1.2 and 1.5) samples confirm the presence of copper, antimony, sulphur and zinc elements in all products obtained at different x (Supporting Information Fig. S6). Table 2 summarizes the EDX results of the Zn series samples. In all the samples, the observed compositions in the Zn-doped-tetrahedrite is close to the expected one, having slightly Sb-rich composition in all samples. Moreover, the compositions of all samples measured by EDX confirm that the substituted Zn atom replaces the Cu sites in the tetrahedrite structure. Figure 8a demonstrates the elemental mapping of the sample at the highest content of Zn (x = 1.5) for Cu Kα, Sb Lα, S Kα and Zn Kα at 20 kV. This indicates a homogeneous spatial distribution of the elements.

**Figure 9:**
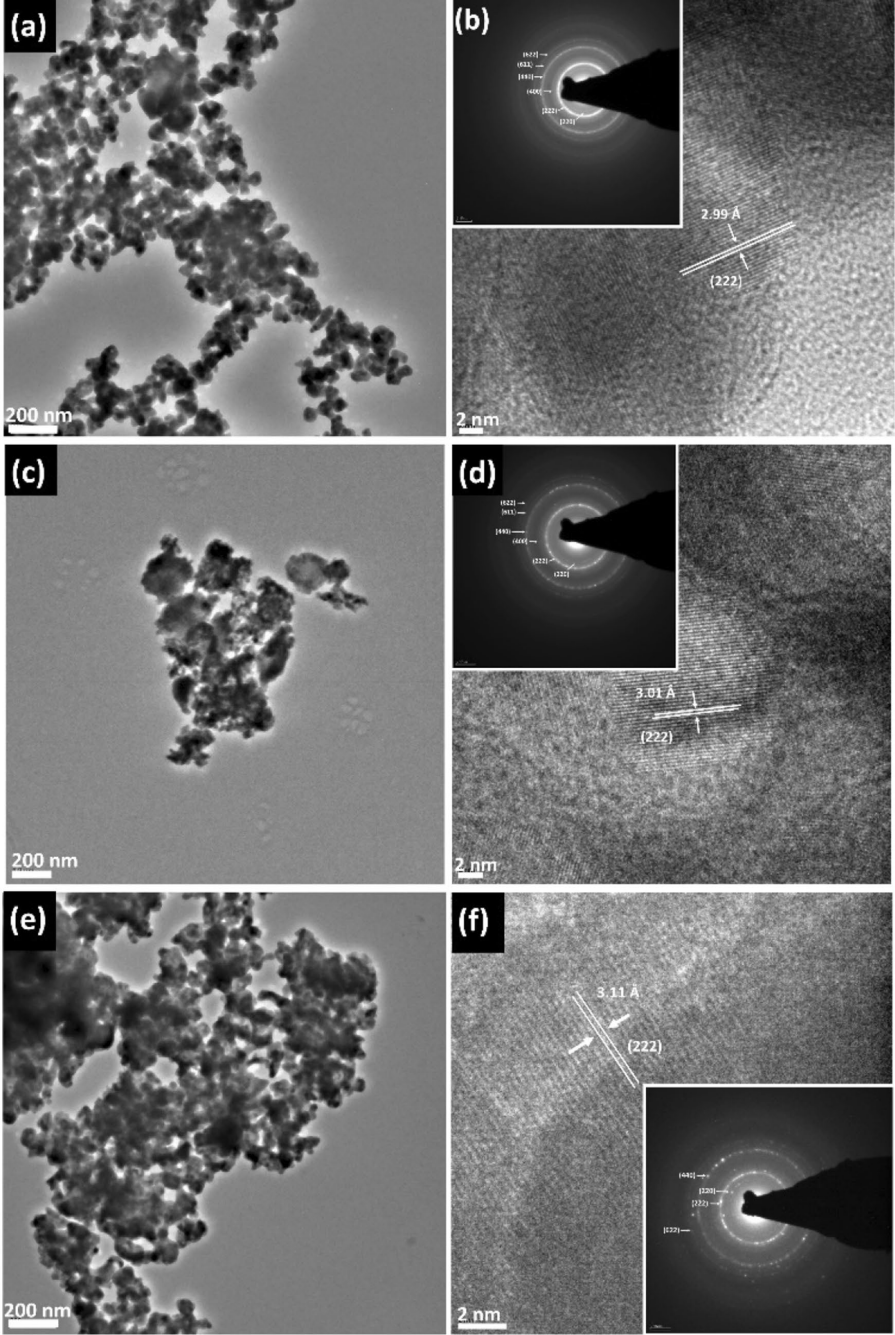
(**a**) TEM image of Cu_12_Sb_4_S_13_ and (**b**) TEM micrograph showing lattice spacings of 2.99 Å for Cu_12_Sb_4_S_13_, was assigned to the (222) plane of the cubic tetrahedrite. Inset in (**b**) shows the corresponding SAED pattern. (**c**) TEM image of Cu_12−x_Zn_x_Sb_4_S_13_ for x = 1.5 and (**d**) TEM micrograph showing lattice spacings of 3.01 Å for Cu_12−x_Zn_x_Sb_4_S_13_, x = 1.5, was assigned to the (222) plane of the cubic tetrahedrite. Inset in (**d**) shows the corresponding SAED pattern. (**e**) TEM image of Cu_12_Sb_4−x_Bi_x_S_13_ for x = 0.5 and (**f**) TEM micrograph showing lattice spacings of 3.11 Å for Cu_12_Sb_4−x_Bi_x_S_13_, x = 0.5, was assigned to the (222) plane of cubic tetrahedrite. Inset in (**f**) shows the corresponding SAED pattern.

TEM and SAED were used in tandem to examine the crystalline structure of the nanoscale Zn-doped tetrahedrite Cu_12−x_Zn_x_Sb_4_S_13_. TEM imaging of Cu_12−x_Zn_x_Sb_4_S_13_ with x = 1.5 (Fig. 9c) reveals that with incorporation the Zn into the tetrahedrite, smaller particles were obtained, with an estimated average size of 28 ± 4 nm, which is consistent with the SEM data^60^. The *d*-spacing from lattice fringes was measured to be 3.01 Å (Fig. 9d), which corresponds to the (222) plane of cubic tetrahedrite, which is slightly larger compared to the *d*-spacing for pure tetrahedrite (2.99 Å). This obtained *d*-spacing implies that, besides slight lattice expansion, no phase change occurred on incorporation of 12 at% Zn into the tetrahedrite structure. The inset of Fig. 9d shows the SAED pattern of the Cu_12−x_Zn_x_Sb_4_S_13_ with x = 1.5 sample. The pattern confirms that the Zn-doped nanomaterials are polycrystalline with the rings indexed to the cubic structure of tetrahedrite, which matches the XRD peaks shown in Fig. 6.

**Figure 10:**
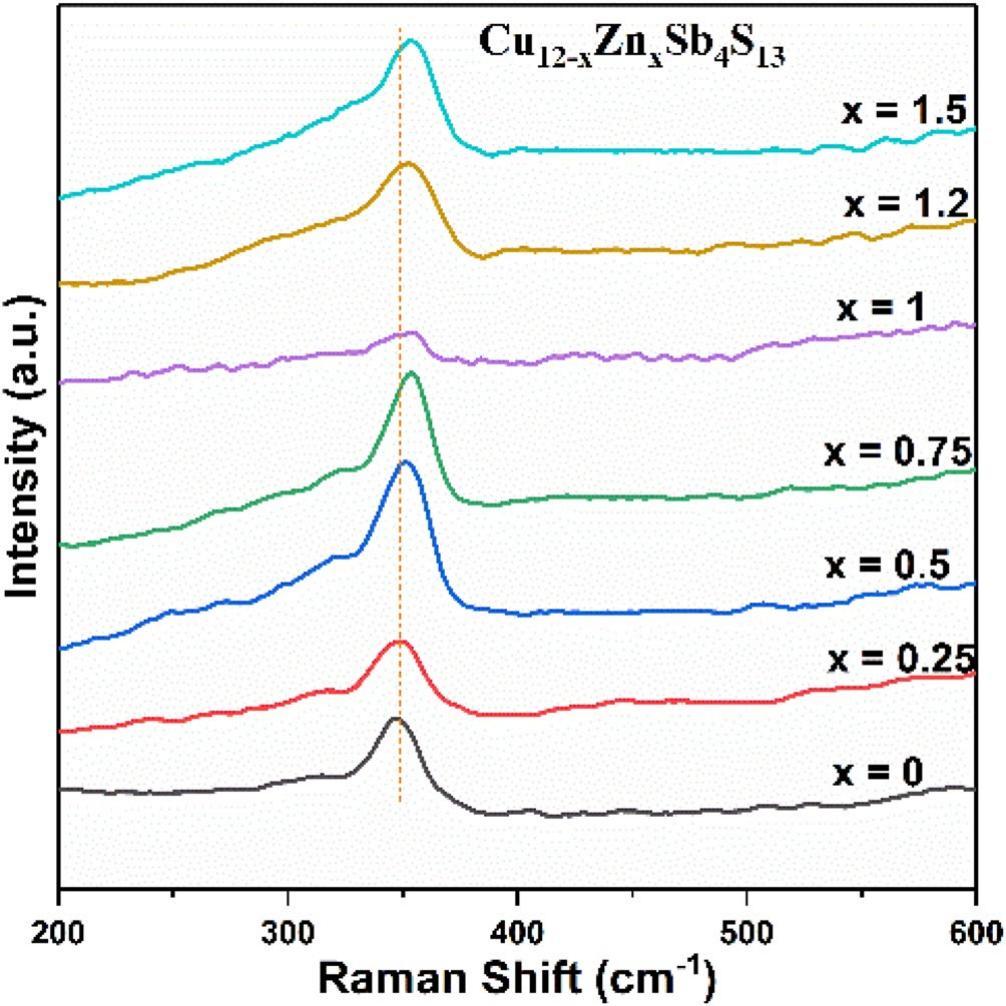
Raman spectra of Zn-doped tetrahedrite Cu_12−x_Zn_x_Sb_4_S_13_ (x = 0.25, 0.5, 0.75, 1, 1.2, 1.5).

Raman spectra of Zn-tetrahedrite Cu_12−x_Zn_x_Sb_4_S_13_ (x = 0.25, 0.5, 0.75, 1, 1.2 and 1.5) samples (Fig. 10) showed a single peak at 350 cm^−1^, which is the characteristic peak of the cubic tetrahedrite phase^63^. Upon increasing the amount of Zn in the tetrahedrite, the Raman peak shifts slightly to higher frequencies from 350 to 355 cm^−1^. This shift in the Raman peak potentially attributed to the change in the lattice vibrations after incorporation of Zn into the tetrahedrite structure. These results are in agreement with a previous study by Bera et al.^75^, which established that the Raman peak position of the Zn-substituted tetrahedrite (Cu_10_Zn_2_Sb_4_S_13_) nanocrystals moves to a higher frequency in the Raman spectra compared to the pure tetrahedrite (Cu_12_Sb_4_S_13_)^75^. Bera and others^75,76^ attribute the shift of these Raman peaks is attributed to the changes in metal—sulfur bond force constants, f_m−s_. The Cu–S bonds are partly replaced by Zn–S during the formation of the Zn-doped tetrahedrite. As a result, a shift to higher frequencies was observed in the Raman spectra because of the f_Zn−S_ ˃ f_Cu−S_, which attributed to the higher mass of zinc in comparison with copper^75,76^.

### Bi^3+^ doped Cu_12_Sb_4_S_13_ nanomaterials

**Figure 11:**
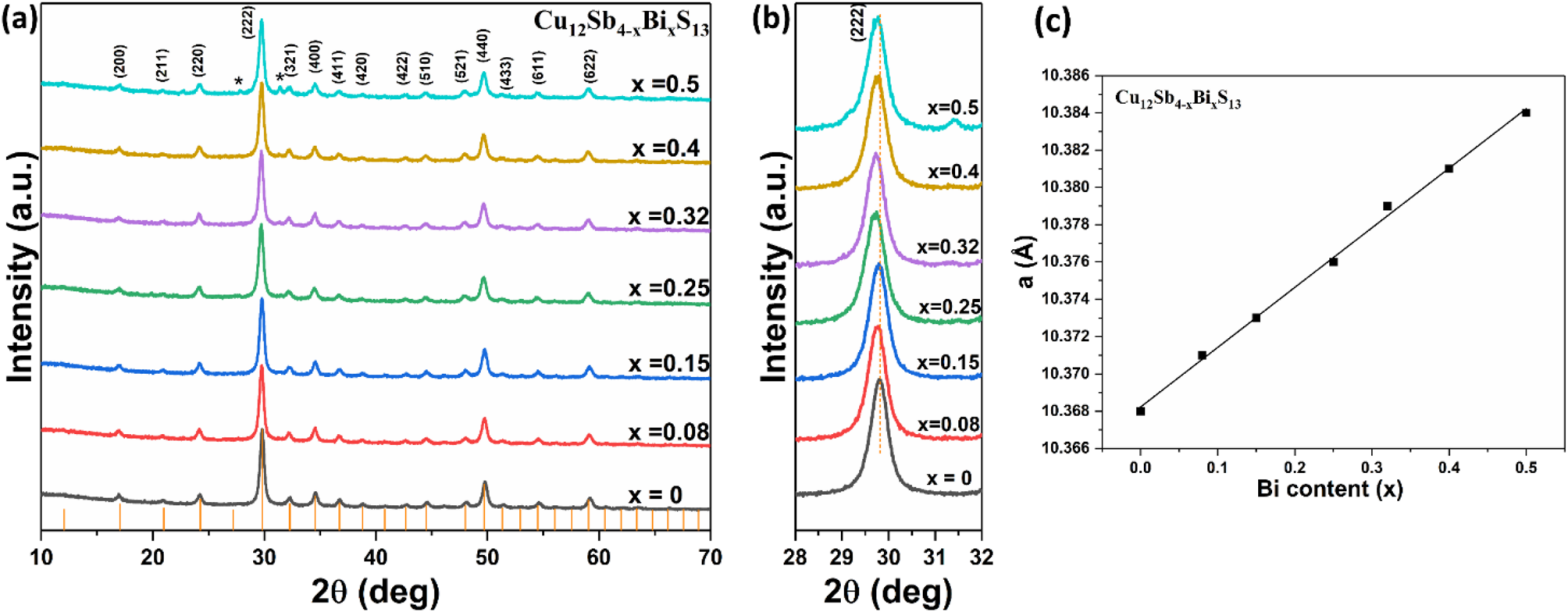
(**a**) p-XRD patterns of Cu_12_Sb_4−x_Bi_x_S_13_ (x = 0.08, 0.15, 0.25, 0.32, 0.4 and 0.5) and (**b**) the maximum intensity peak (222) of Bi-doped tetrahedrite samples. Orange lines are from standard ICDD: 01-088-0283 for cubic Cu_12_Sb_4_S_13_ (tetrahedrite). (**c**) Lattice parameter a as a function of Bi composition.

Bi doped tetrahedrite nanomaterials were prepared by using the optimized quantities of Cu and Sb xanthate precursors (i.e. 2:1 ratio). p-XRD shows that Cu_12_Sb_4−x_Bi_x_S_13_ (x = 0.08, 0.15, 0.25, 0.32, 0.4 and 0.5) nanomaterials also exhibit the cubic tetrahedrite structure (Cu_12_Sb_4_S_13_ ICDD: 01-088-0283) with, in the main, no secondary phases observed (Fig. 11a). Only the sample at the Bi content of x = 0.5 (12%) shows two minor additional peaks corresponding to Bi_2_S_3_ (as indicated by asterisks), implying that this value is above the solubility limit of Bi in Cu_12_Sb_4_S_13_. As the Bi content increases in the samples, the most intense diffraction peak, which corresponds to the (222) plane is gradually shifted towards lower angles, as can be seen in Fig. 11b. In addition, the other two peaks indexed to the (440) and (662) planes exhibit a shift towards lower angle as the Bi dopant concentration increased in the samples (Supporting Information Fig. S7). This shift indicates an expansion in the crystal structure. The lattice parameters *a* of Cu_12_Sb_4−x_Bi_x_S_13_ samples with various doping contents were calculated from the X-ray-diffraction data (Table 3) and were found to increase linearly with increasing the Bi content in the samples, as shown in Fig. 11c. This increase in the lattice parameter confirms the substitution of Bi at Sb sites, since ionic radii for Bi^3+^ (0.96 Å) is slightly larger than that for Sb^3+^ (0.76 Å)^71^. Similar behaviour was reported by Kumar et al.^77^ for the Bi-doped samples. In this instance the lattice parameter *a* increases from 10.326 Å for undoped tetrahedrite to 10.375 Å for Bi-doped tetrahedrite Cu_12_Sb_4−x_Bi_x_S_13_ with x = 0.8. The changes in the lattice parameter values for the Bi-doped samples are larger than that of the Zn-doped samples. This is a result of the radius difference between Bi (0.96 Å) and Sb (0.76 Å) which is larger than that between Cu (0.6 Å) and Zn (0.68 Å) (see Supporting Information Fig. S8). This observation is consistent with the previous study by Kumar et al.^55^. They compared the lattice parameters for various dopants at Cu site i.e. Cu_12−x_M_x_Sb_4_S_13_, (where M = Ni, Cd, Co, Zn, Mn) at x = 1.5. They found that as the ionic radii of these doping elements increased (Ni < Co < Zn < Mn < Cd), expansion in the crystal structure takes place, causing an increase in the lattice parameters (follow the trend Ni < Co < Zn < Mn < Cd)^55^.

**Table 3:** Lattice parameters a, EDX compositional analysis and the estimated band gap (E_g_) of Cu_12_Sb_4−x_Bi_x_S_13_ (x = 0.08, 0.15, 0.25, 0.32, 0.4 and 0.5).

X	Composition	Lattice parameters a (Aͦ)	Elemental composition by EDX (atomic%)				E_g_ (eV)
			Cu	Sb	Bi	S	
0	Cu_12_Sb_4_S_13_	10.368	41.1	14.2	0	44.6	1.49
0.08	Cu_12_Sb_3.92_Bi_0.08_ S_13_	10.371	40.8	14.3	0.5	44.4	1.53
0.15	Cu_12_Sb_3.84_Bi_0.16_ S_13_	10.373	40.4	13.8	0.8	45.0	1.56
0.25	Cu_12_Sb_3.76_Bi_0.24_ S_13_	10.376	40.7	14.6	0.9	43.8	1.58
0.32	Cu_12_Sb_3.68_Bi_0.32_ S_13_	10.379	40.5	13.9	1.2	44.4	1.59
0.4	Cu_12_Sb_3.6_Bi_0.4_ S_13_	10.381	40.9	14.2	1.5	43.4	1.66
0.5	Cu_12_Sb_3.52_Bi_0.48_ S_13_	10.384	40.6	13.8	1.8	43.8	1.72

The SEM images of the Bi-doped tetrahedrite Cu_12_Sb_4−x_Bi_x_S_13_ samples with different Bi content are illustrated in Fig. S9 (Supporting Information). The morphologies of all the Bi doped samples is similar to that of the pure tetrahedrite. Limited differences were observed as the Bi content increased in the sample. The particle size of the sample at the highest content of Bi (x = 0.5) was found to be in the range of 62 ± 9, which is very similar to that of the undoped tetrahedrite (61.2 ± 8). The EDX spectra of the Cu_12_Sb_4−x_Bi_x_S_13_ indicated the characteristic peaks of copper, antimony, sulphur and bismuth in all samples obtained at different x and no impurities were observed (Supporting Information Fig. S6). Table 3 summarizes the EDX results of the Bi series samples. In all the samples, the observed compositions in the Bi-doped-tetrahedrite is close to the expected values. Figure 8b shows EDX elemental mapping of the sample at the highest content of Bi (x = 0.5) for the Cu Kα, Sb Lα, S Kα and Bi Mα emissions, which indicate a homogeneous distribution of the elements at the scale investigated.

The structure of the Cu_12_Sb_4−x_Bi_x_S_13_ with x = 0.5 sample was further investigated by using TEM and SAED. The images collected are displayed in Fig. 9e,f. The TEM image of this sample shows similar particle sizes to that of pure tetrahedrite, with an estimated average size of 62 ± 7 nm. This is consistent with the SEM data discussed previously (Fig. 9e). The d-spacing for the (222) plane of the Cu_12_Sb_4−x_Bi_x_S_13_ for x = 0.5 was 0.311 nm (Fig. 9f) which is larger than that for pure tetrahedrite (0.302 nm) due to bismuth incorporation. This increase in the *d*-spacing may be caused by both the higher mass of Bi and the longer Bi–S distances compared with Sb^78^. The SAED pattern of the Cu_12_Sb_4−x_Bi_x_S_13_ with x = 0.5 nanomaterial (inset of Fig. 9f) displays clear ordered diffraction rings, corresponding to the (220), (222), (440) and (622) lattice planes of the cubic tetrahedrite, which agree well with the XRD pattern in Fig. 11.

**Figure 12:**
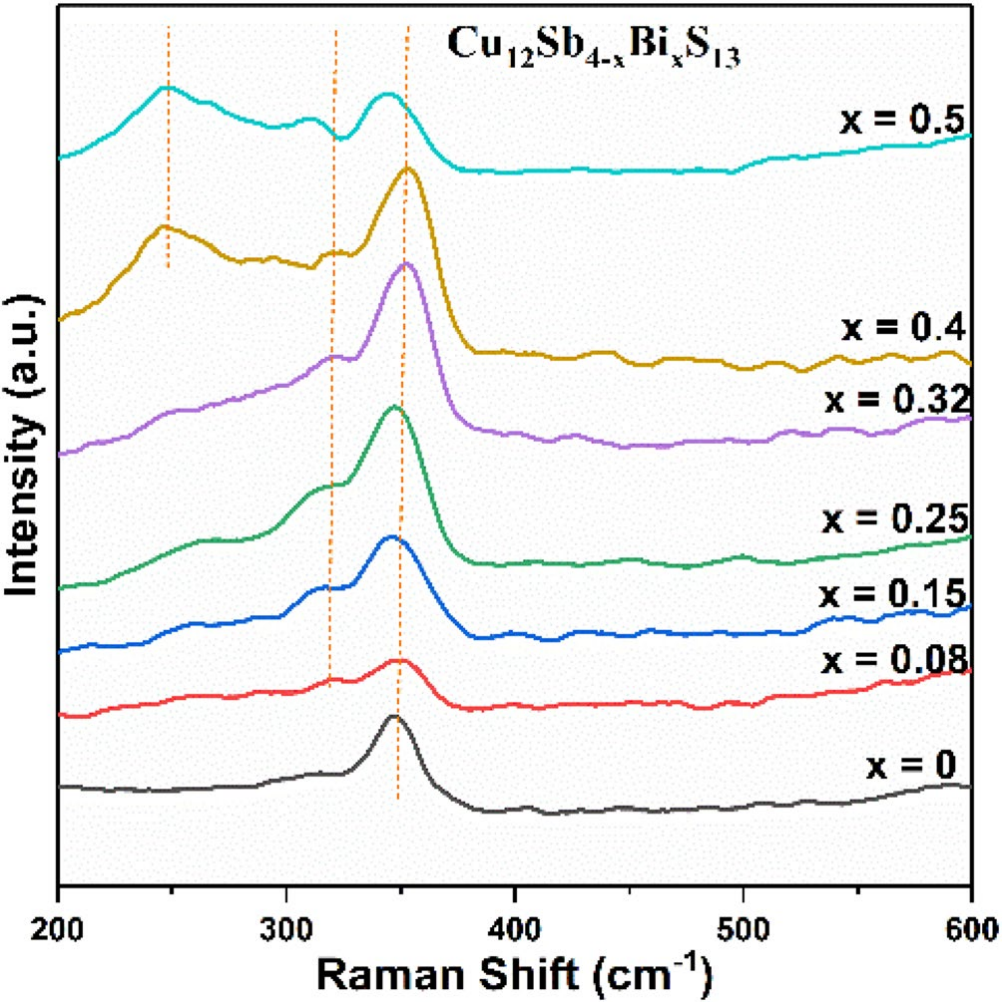
Raman spectra of Bi-doped tetrahedrite Cu_12_Sb_4−x_Bi_x_S_13_ (x = 0.08, 0.15, 0.25, 0.32, 0.4 and 0.5).

The Raman spectra for all the Bi-doped tetrahedrite Cu_12_Sb_4−x_Bi_x_S_13_ (x = 0.08, 0.15, 0.25, 0.32, 0.4 and 0.5) samples are shown in Fig. 12. A broad band is observed at approximately at 351 cm^−1^ for all the samples, which is the characteristic peak of the cubic tetrahedrite phase^63^. Also, a peak begins to emerge at 319 cm^−1^ for the x = 0.08 sample, which assigned to the symmetric bending mode of the cubic tetrahedrite. This new peak shifts towards lower frequencies as the Bi content is increased. The absence of this peak in the pure tetrahedrite sample might be attributed to the poor crystallinity of the sample or the peak was too weak to be identified^28^. In addition, another peak begins to appear at 250 cm^−1^ when the Bi content increased to x = 0.4, which corresponds to the B_1g_ anti-symmetric stretching mode for orthorhombic Bi_2_S_3_^40,79^. However, in the XRD data, the presence of Bi_2_S_3_ peaks is found only at the Bi content of x = 0.5 (12%) sample which highlights the importance of a multiple technique approach to characterisation for these materials. The shift of the peaks towards lower wavenumbers were detected as the amount of Bi increased in the samples. This may be attributed to the higher mass of Bi and the longer Bi–S distance in contrast with Sb^79,80^.

### Optical band gap

For optical bandgap measurements, thin-film samples of chalcostibite, tetrahedrite and Zn/Bi-doped tetrahedrite were prepared by doctor bladed using a slurry of Cu, Sb, Zn or Bi xanthates in hexane and deposited on a glass substrate. The deposited precursors were heat treated in a quartz tube under a flow of nitrogen at 250 °C for 1 hour. The films were characterized by p-XRD and EDX spectroscopy to confirm the formation of desired phases and the diffraction patterns indicated that the deposited films have exact same phase as obtained from the powdered products (Supporting Information Fig. S10). The elemental composition of the films was determined by EDX spectroscopy and the stoichiometry of the films was comparable to the powdered films (Supporting Information Table S1–S3). The surface analysis of the films indicated that the deposited films were not of good quality and were full of cracks (Supporting Information Fig. S11, S12). The adherence to the glass substrate was not strong, and the particulate material can be easily scratched off from the substrate. The probable reasons for low quality of the films may be high volatility of hexane, leaving the slurry dried on the substrate and the escape of volatile gases (COH and ethene) during the thermal decomposition of the xanthate complexes, may have resulted in the formation of cracked surface and loosely bound films.

**Figure 13:**
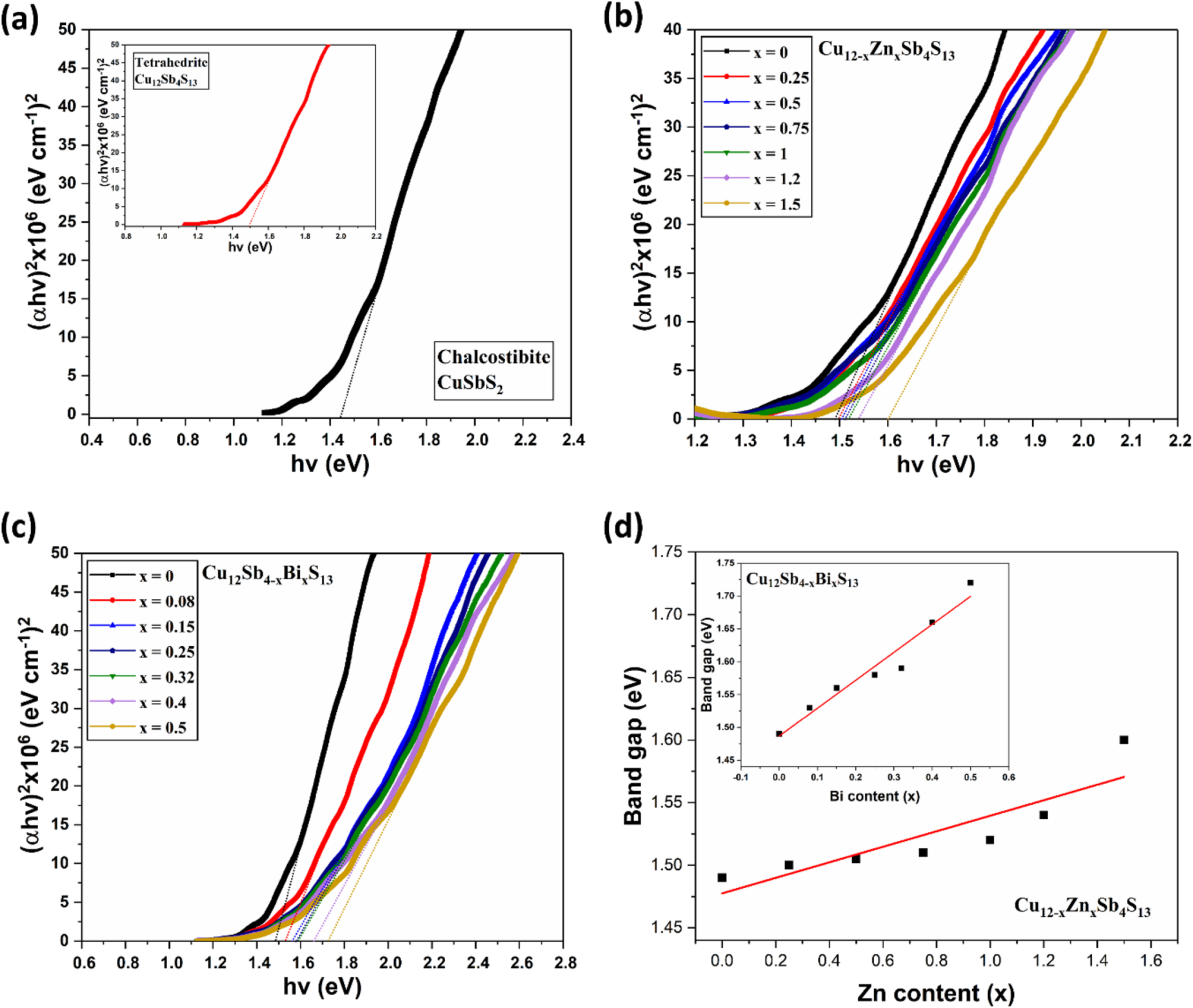
Estimation of optical band gap in Cu-Sb-S materials. Tauc plots for the (**a**) CuSbS_2_ and Cu_12_Sb_4_S_13_ (inset), (**b**) Cu_12−x_Zn_x_Sb_4_S_13_ (x = 0.25, 0.5, 0.75, 1, 1.2, 1.5) and (**c**) Cu_12_Sb_4−x_Bi_x_S_13_ (x = 0.08, 0.15, 0.25, 0.32, 0.4 and 0.5). (**d**) Variation of the band gap for Cu_12−x_Zn_x_Sb_4_S_13_ samples as a function x_Zn_, and the inset shows the variation of the band gap for Cu_12_Sb_4−x_Bi_x_S_13_ samples as a function x_Bi_.

Optical absorption spectra of these samples were recorded in the wavelength range of 400–1100 nm (Supporting Information Fig. S13a,b). The band gap of the films can be calculated using the formula (αhν)^n^ = A(*h*ν – Eg): Eg is the optical band gap, *h*ν is the photon energy, α is the absorption coefficient, A is a constant characteristic of the material, and n = 2, ½ for allowed direct and allowed indirect transitions, respectively. Chacostibite CuSbS_2_ and tetrahedrite Cu_12_Sb_4_S_13_ ternary phases are reported as direct band gap materials^30,81^. (Note: Some reports have also indicated the presence of both direct and indirect band gaps for CuSbS_2_ phase^6^, and the probable reason is that CuSbS_2_ exists in layered structure and it has been shown that the band gap of layered semiconducting materials can be changed from indirect to direct by tuning the thickness of the nanosheets^82^) Furthermore, plots of the (αhν)^n^ versus hν for n = 2 and ½ show a linear behaviour for n = 2, which confirm the presence of a direct transition in both phases. Figure 13a–c present the estimation of band gaps using the Tauc plots for all Cu–Sb–S samples. For the chacostibite nanomaterials, the direct band gaps were estimated to be 1.44 eV; very close to the reported values for this material^9,47,83^. The value of the band gap for the pure tetrahedrite was estimated to be 1.49 eV, which was also close to the reported value^15,31^. For the Cu_12−x_Zn_x_Sb_4_S_13_, the band gaps are estimated to be ∼ 1.49, 1.5, 1.505, 1.51, 1.52, 1.54, and 1.6 eV, respectively, as the Zn content increased in the films from x = 0 to x = 1.5. A similar observation has been reported by Bera et al.^75^, as they found the energy band gap of the Zn-substituted tetrahedrite (Cu_10_Zn_2_Sb_4_S_13_) nanocrystals increased compared to the pure tetrahedrite (Cu_12_Sb_4_S_13_) nanocrystals. Similarly, the band gaps of Cu_12_Sb_4−x_Bi_x_S_13_ samples are estimated to be ∼1.49, 1.53, 1.56, 1.58, 1.59, 1.66, and 1.72 eV, respectively, with increasing Bi content in the films from x = 0 to x = 0.5. Figure 13d shows the variation of the band gap of the Zn and Bi-doped samples as a function of x, demonstrating that the band gap of both doped samples increases with increasing dopant concentration. The increase of the band gap with increasing dopant into the tetrahedrite was significant in Bi doped samples compared to Zn-doped samples (see Supporting Information Fig. S14).

## Conclusions

Toward the synthesis of chalcostibite (CuSbS_2_) and tetrahedrite (Cu_12_Sb_4_S_13_), the use of the Cu (II) xanthate (Cu[S_2_COEt]_2_) and Sb (III) xanthate (Sb[S_2_COEt]_3_) has been explored as the Cu, Sb and S sources for the desired ternary copper antimony sulfide nanomaterials. The compositions of the obtained materials were adjusted by varying the molar ratio of the copper and antimony xanthate precursors. Powder XRD and Raman spectroscopy indicates that using the expected ratios of Cu and Sb precursors for the pure tetrahedrite synthesis (3:1 ratio) yielded an extra binary copper sulfide impurity phase, while using less amount of Cu precursor (2:1 ratio) produced a single-phase tetrahedrite without the presence of any impurities. Zn-doped tetrahedrites Cu_12−x_Zn_x_Sb_4_S_13_ (x = 0.25, 0.5, 0.75, 1, 1.2 and 1.5) and Bi-doped tetrahedrites Cu_12_Sb_4−x_Bi_x_S_13_ (x = 0.08, 0.15, 0.25, 0.32, 0.4 and 0.5) were also synthesized using Zn(II) xanthate (Zn[S_2_COEt]_2_) and Bi(III) xanthate (Bi[S_2_COEt]_3_) as the Zn and Bi sources. P-XRD analysis confirm the presence of cubic tetrahedrite for all two doped series. The only exception was for Cu_12_Sb_4−x_Bi_x_S_13_ with x = 0.5, which showed a secondary phase, implying that this value is above the solubility limit of Bi in Cu_12_Sb_4_S_13_ (12%). We found that the lattice parameters *a* in both tetrahedrite samples with Zn- and Bi-doping increased with increasing dopant concentration. SEM images indicated the formation of smaller particle sizes with Zn incorporation into the tetrahedrite, whereas Bi doped samples exhibited a similar particle size to that of pure tetrahedrite. The estimated band gap of Cu_12−x_Zn_x_Sb_4_S_13_ films varied from 1.49 to 1.6 eV, while the band gap of Cu_12_Sb_4−x_Bi_x_S_13_ films increased from 1.49 to 1.72 eV with increasing x. The effect of doping into the tetrahedrite on the lattice parameter and band gap energy was more significant in Bi-doped samples compared to Zn-doped samples. Optical measurements of the films suggest that CuSbS_2_ and Cu_12_Sb_4_S_13_ have direct band gaps of 1.44 and 1.49 eV respectively. This enables them to offer the most promising properties for use in solar energy conversion applications. The tuning of the magnitude of the band gap energy in these materials, and thus control over carrier concentration, is also interesting for thermoelectric applications. Overall, our approach represents a rapid, scalable, low temperature process toward these interesting materials.

## Supplementary Information

Supplementary Information.
