# Supplementary material for: Scalable synthesis of Cu–Sb–S phases from reactive melts of metal xanthates and effect of cationic manipulation on structural and optical properties
Source: Sci Rep. 2021 Jan 21;11:1887. doi: 10.1038/s41598-020-80951-5 (PMC7820284; doi:10.1038/s41598-020-80951-5)
Supplement: Supplementary file 1 — Supplementary Information. [file 41598_2020_80951_MOESM1_ESM.docx]

**Supplementary Information**

Scalable synthesis of Cu-Sb-S phases from reactive melts of metal xanthates and effect of cationic manipulation on structural and optical properties

Tahani Alqahtani,^a^ Malik Dilshad Khan,*^b,c^ David J. Lewis,^c^* Xiang Li Zhong,^d^ and Paul O’Brien^#^

^a^ School of Physics, Imam Abdulrahman Bin Faisal University, Dammam, Saudi Arabia.

^b^ Department of Chemistry, University of Zululand, Private bag X1001, Kwa-Dlangezwa 3880, South Africa.

^c^ School of Materials, The University of Manchester, Oxford Road, Manchester, M13 9PL, UK.

# Dedicated to Prof. O’Brien, who passed away during the preparation of this paper.

*E-mail: [david.lewis-4@manchester.ac.uk](mailto:david.lewis-4@manchester.ac.uk); [malikdilshad@hotmail.com](mailto:malikdilshad@hotmail.com)





Figure S1: TGA plots of xanthate complexes of Sb^III^, Cu^II^, Zn^II^ and Bi^III^.


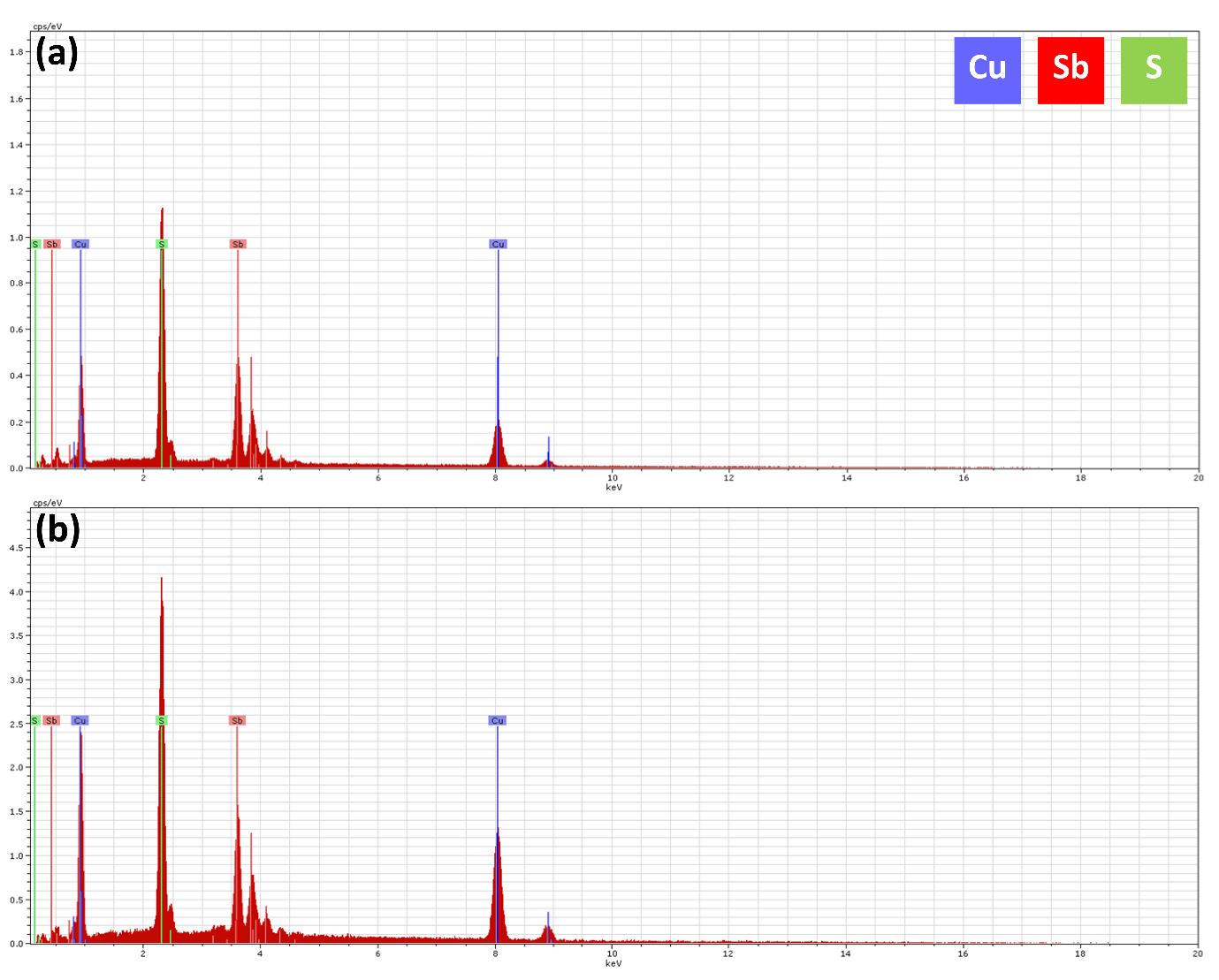


Figure S2: EDX spectra of (a) chalcostibite CuSbS_2_ (b) tetrahedrite Cu_12_Sb_4_S_13_.


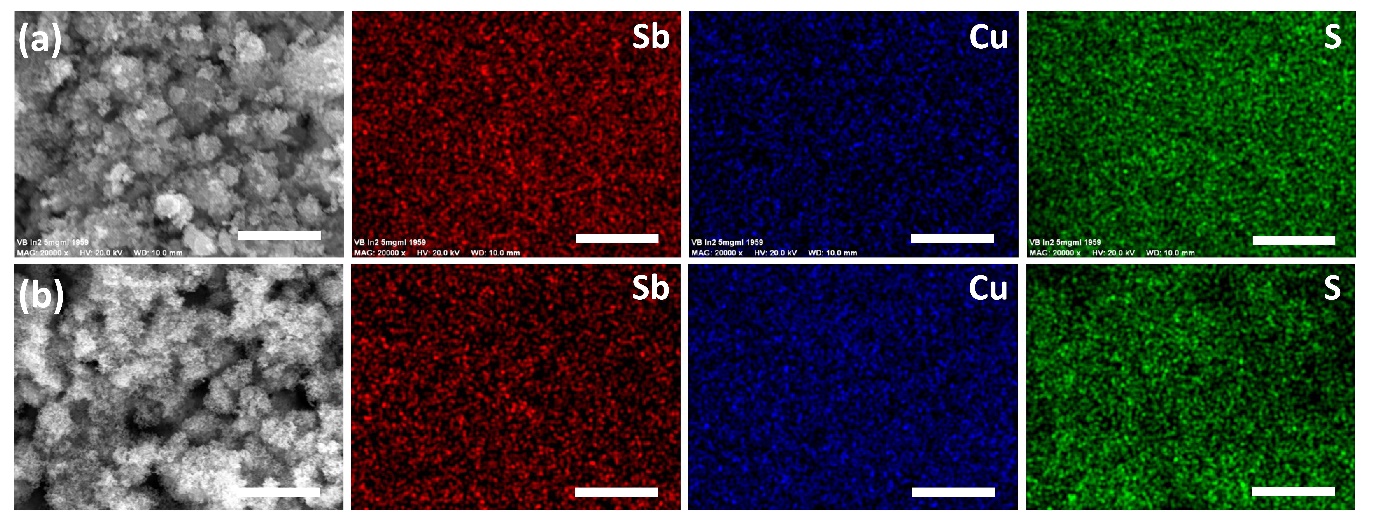


Figure S3: EDX elemental mapping of (a) chalcostibite CuSbS_2_ (b) tetrahedrite Cu_12_Sb_4_S_13_. Scale bars = 5 µm.


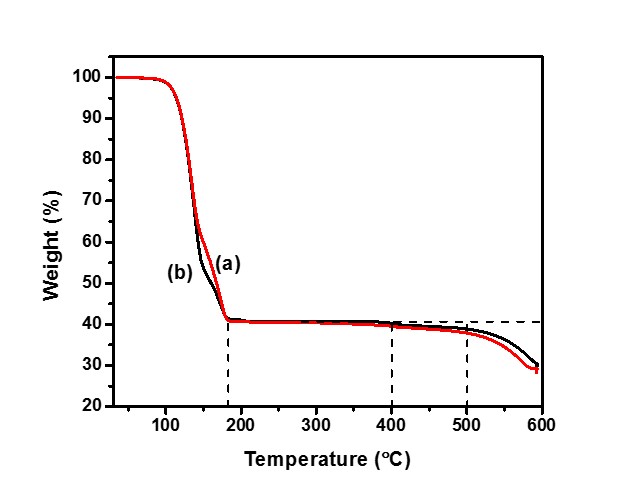


Figure S4: TGA plots of mixture of xanthate complexes in (a) 3:1 and (b) 2:1, to prepare tetrahedrite phase.


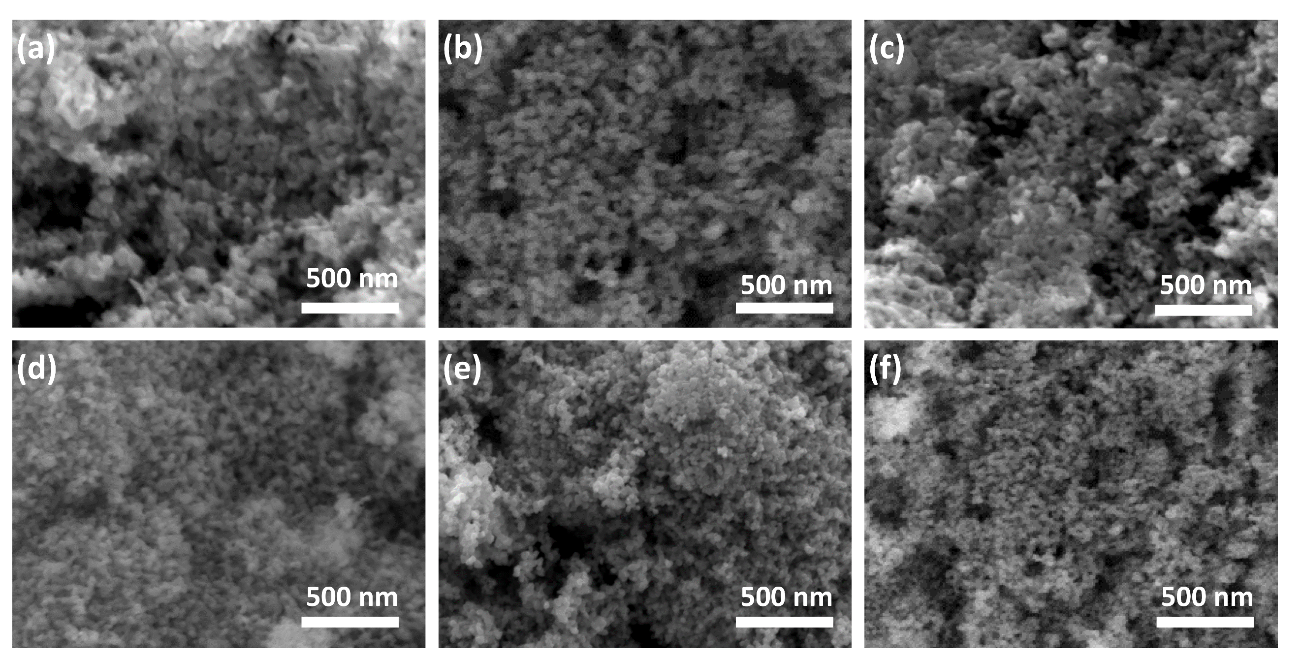


Figure S5: SEM images of Cu_12-x_Zn_x_Sb_4_S_13_ nanostructures with different Zn content (a) x = 0.25, (b) x = 0.5, (c) x = 0.75, (d) x = 1, (e) x = 1.2 and (f) x = 1.5.


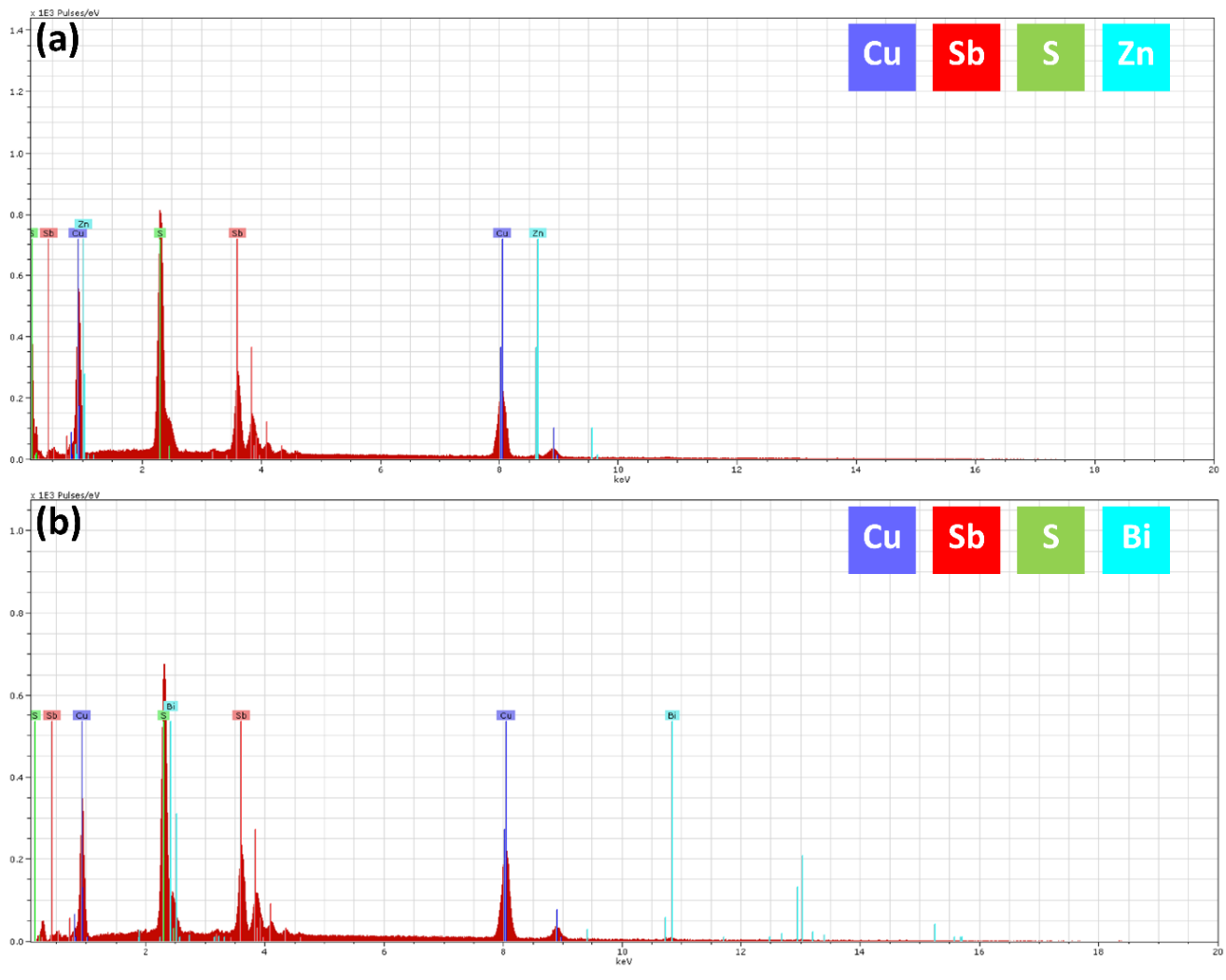


Figure S6: EDX spectra of (a) Cu_12-x_Zn_x_Sb_4_S_13_ (x = 1.5) and (b) Cu_12_Sb_4-x_Bi_x_S_13_ (x = 0.5).


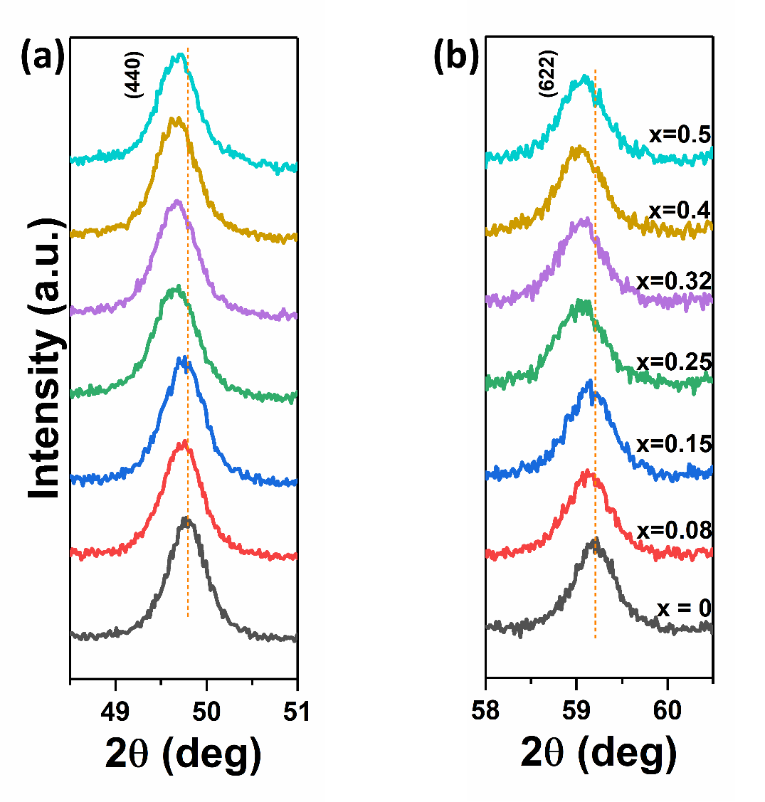


Figure S7: Magnification of (440) and (622) diffraction peaks of Cu_12_Sb_4-x_Bi_x_S_13_ (x = 0.08, 0.15, 0.25, 0.32, 0.4 and 0.5).





Figure S8: Comparation of the lattice constants a of Cu_12-x_Zn_x_Sb_4_S_13_ and Cu_12_Sb_4-x_Bi_x_S_13_ with different dopant percentage (2-12%).


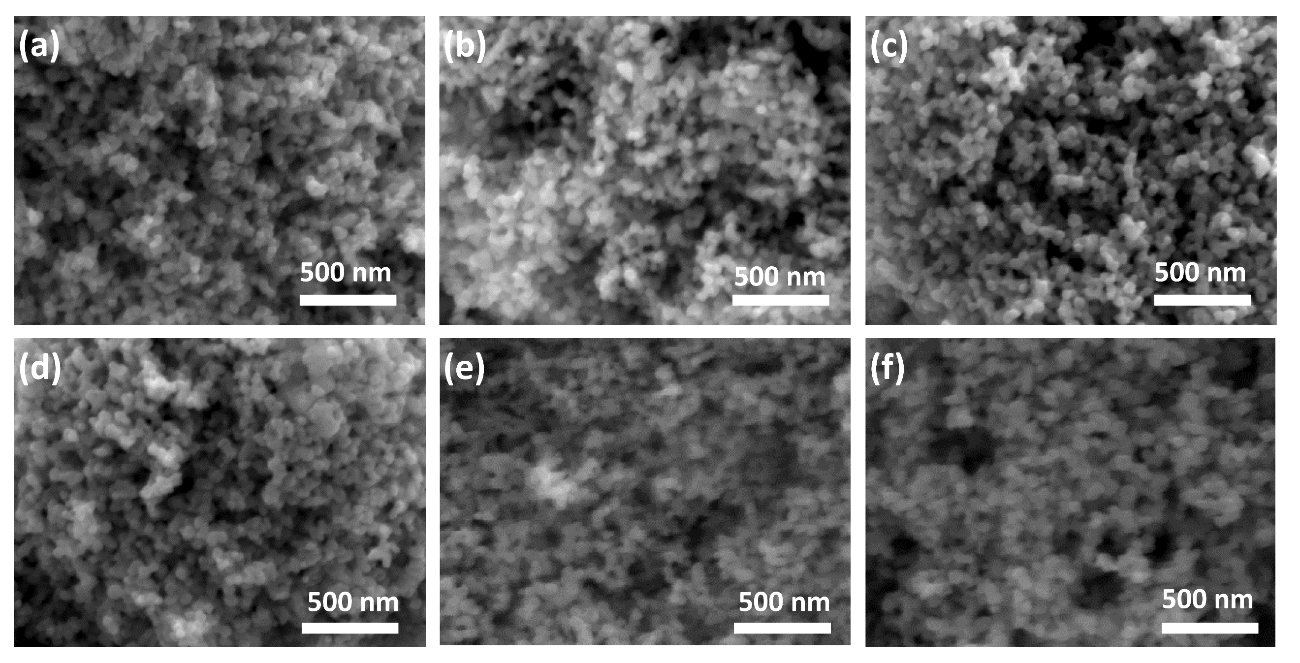


Figure S9: SEM images of the Cu_12_Sb_4-x_Bi_x_S_13_ samples with different Bi content (a) x = 0.08, (b) x = 0.15, (c) x = 0.25, (d) x = 32, (e) x = 0.4 and (f) x = 0.5.


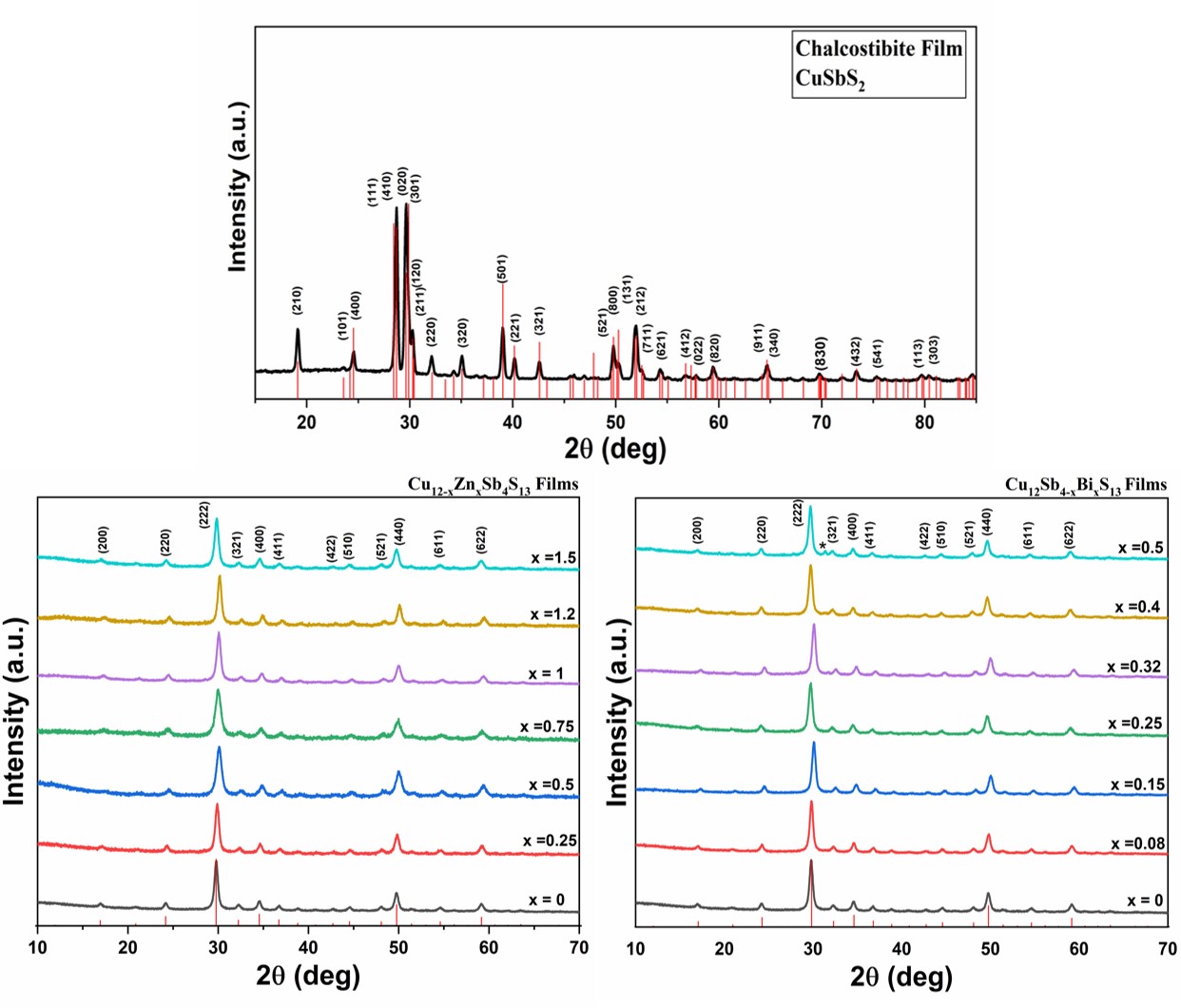


Figure S10: P-XRD analysis of chalcostibite, Cu_12-x_Zn_x_Sb_4_S_13_ (x = 0.25, 0.5, 0.75, 1, 1.2, 1.5) and Cu_12_Sb_4-x_Bi_x_S_13_ (x = 0.08, 0.15, 0.25, 0.32, 0.4 and 0.5) films deposited by Dr. Blade method.

Table S1. The content of Cu, Sb and S in chalcostibite film.

| Sample | Cu (at%) | Sb (at%) | S (at%) |
| --- | --- | --- | --- |
| CuSbS_2_ | 30.1 | 21.0 | 48.9 |

*Table S2. EDX compositional analysis of Cu_12-x_Zn_x_Sb_4_S_13_ films (x = 0.25, 0.5, 0.75, 1, 1.2, 1.5).*

| X | Composition |  | Elemental composition by EDX (atomic%) | | | |
| --- | --- | --- | --- | --- | --- | --- |
|  |  |  | Cu | Zn | Sb | S |
| 0 | Cu_12_Sb_4_S_13_ |  | 41.2 | 0 | 15.5 | 43.3 |
| 0.25 | Cu_11.76_Zn_0.24_ Sb_4_S_13_ |  | 38.1 | 0.6 | 14.4 | 46.9 |
| 0.5 | Cu_11.52_Zn_0.48_ Sb_4_S_13_ |  | 36.9 | 1.9 | 16.3 | 45.0 |
| 0.75 | Cu_11.28_Zn_0.72_ Sb_4_S_13_ |  | 38.1 | 2.0 | 15.9 | 44.8 |
| 1 | Cu_11.04_Zn_0.96_ Sb_4_S_13_ |  | 38.0 | 2.9 | 15.5 | 43.6 |
| 1.2 | Cu_10.8_Zn_1.2_ Sb_4_S_13_ |  | 35.8 | 3.1 | 15.6 | 45.5 |
| 1.5 | Cu_10.56_Zn_1.44_ Sb_4_S_13_ |  | 35.8 | 4.3 | 15.3 | 44.6 |

Table S3. EDX compositional analysis of Cu_12_Sb_4-x_Bi_x_S_13_ films (x = 0.08, 0.15, 0.25, 0.32, 0.4 and 0.5).

| X | Composition |  | Elemental composition by EDX (atomic%) | | | |
| --- | --- | --- | --- | --- | --- | --- |
|  |  |  | Cu | Sb | Bi | S |
| 0 | Cu_12_Sb_4_S_13_ |  | 41.2 | 15.5 | 0 | 43.3 |
| 0.08 | Cu_12_Sb_3.92_Bi_0.08_ S_13_ |  | 38.8 | 14.3 | 0.6 | 46.3 |
| 0.15 | Cu_12_Sb_3.84_Bi_0.16_ S_13_ |  | 40.0 | 14.6 | 0.7 | 44.6 |
| 0.25 | Cu_12_Sb_3.76_Bi_0.24_ S_13_ |  | 38.2 | 14.7 | 0.9 | 46.1 |
| 0.32 | Cu_12_Sb_3.68_Bi_0.32_ S_13_ |  | 38.7 | 15.1 | 1.3 | 44.8 |
| 0.4 | Cu_12_Sb_3.6_Bi_0.4_ S_13_ |  | 38.5 | 15.1 | 1.6 | 44.8 |
| 0.5 | Cu_12_Sb_3.52_Bi_0.48_ S_13_ |  | 39.8 | 13.1 | 1.9 | 45.1 |


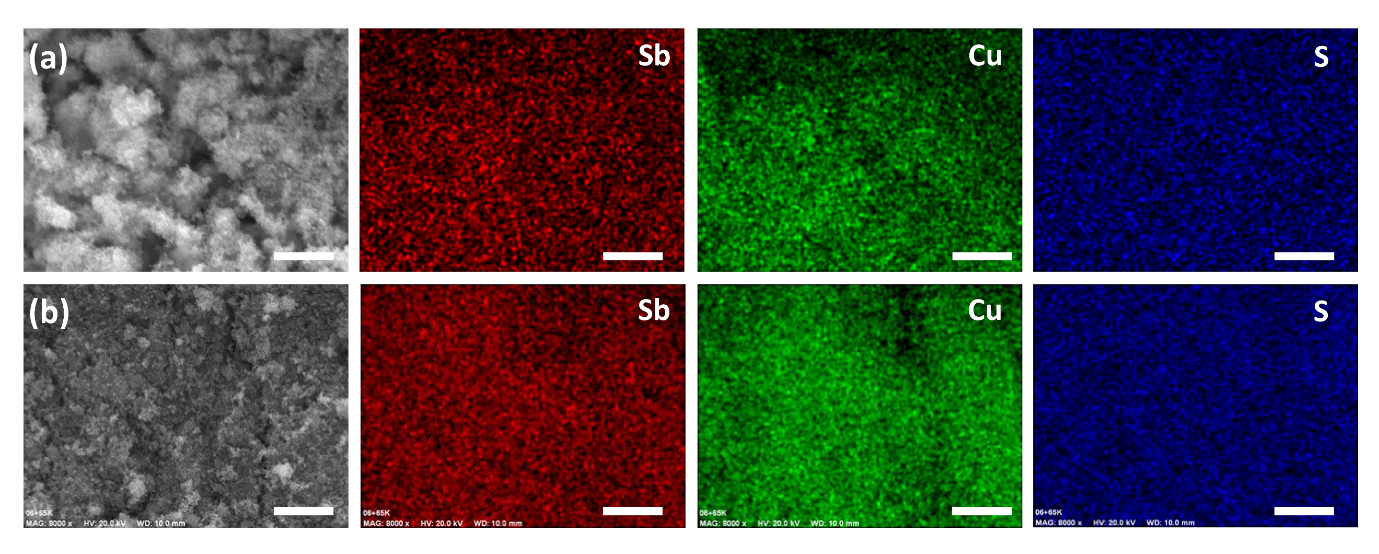


Figure S11: EDX elemental mapping of (a) chalcostibite CuSbS_2_ films (b) tetrahedrite Cu_12_Sb_4_S_13_ films. Scale bars = 10 µm.


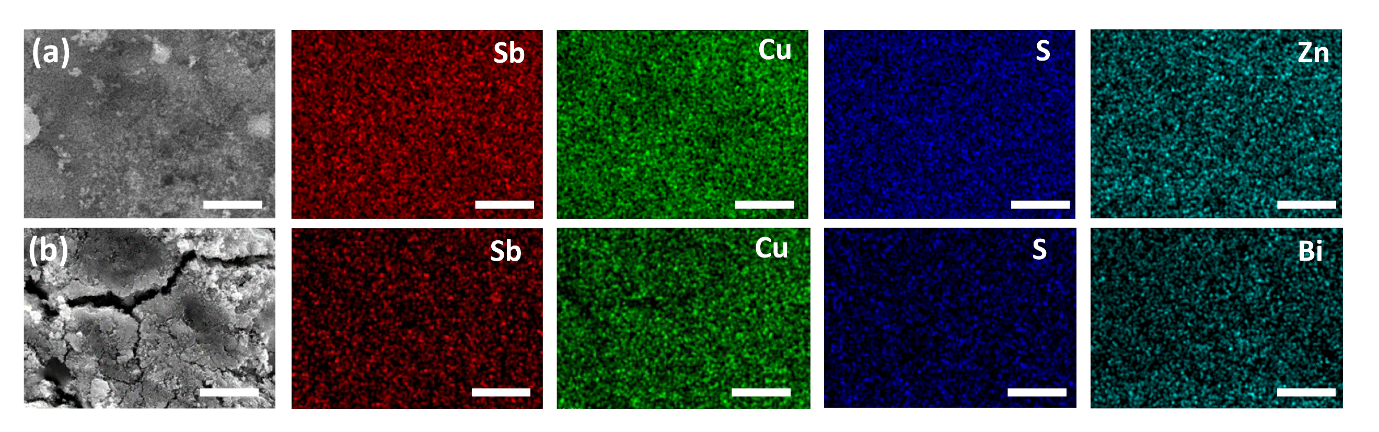


Figure 12: Representative EDX elemental mapping (20 kV) of Cu Kα, Sb Lα, S Kα and Zn Kα/Bi Mα for (a) Cu_12-x_Zn_x_Sb_4_S_13_ (x = 1.5) and (b) Cu_12_Sb_4-x_Bi_x_S_13_ (x = 0.5). Scale bars = 10 µm.


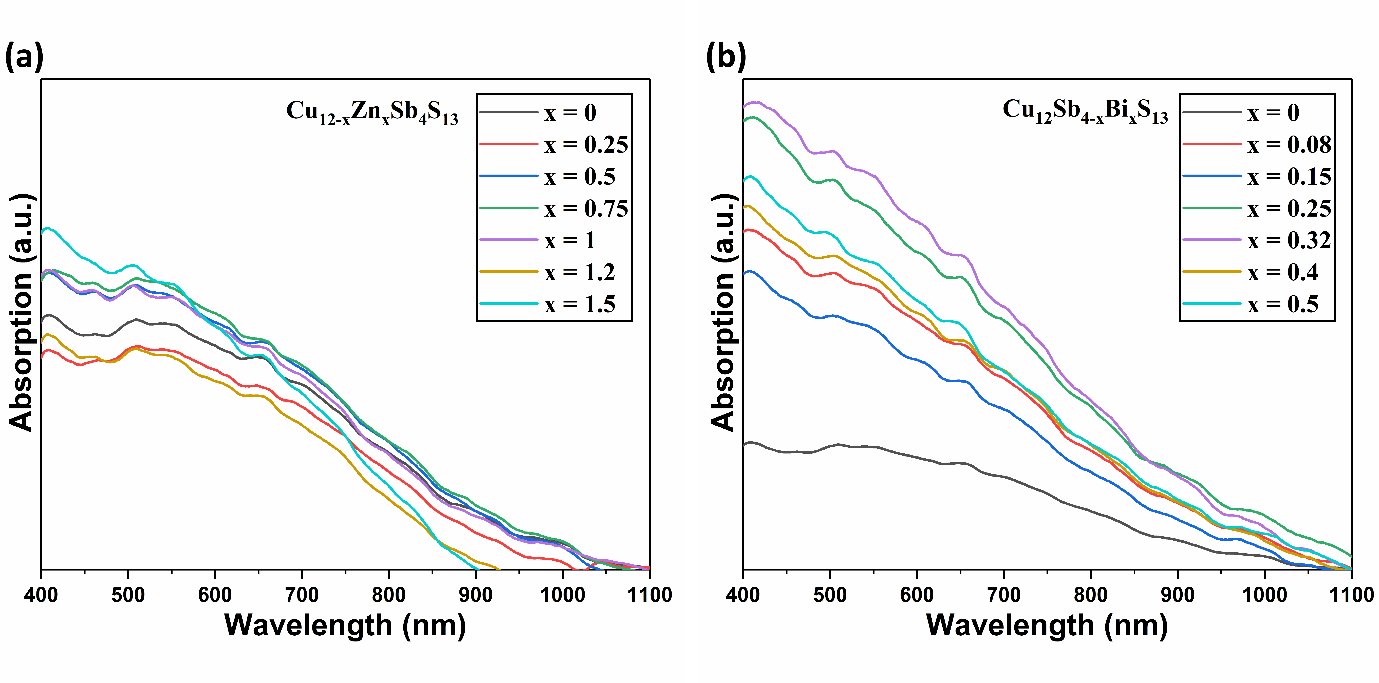


Figure S13: Absorption spectra of (a) Cu_12-x_Zn_x_Sb_4_S_13_ (x = 0.25, 0.5, 0.75, 1, 1.2, 1.5) and (b) Cu_12_Sb_4-x_Bi_x_S_13_ (x = 0.08, 0.15, 0.25, 0.32, 0.4 and 0.5).





Figure S14: Comparation of the band gap changes of Cu_12-x_Zn_x_Sb_4_S_13_ and Cu_12_Sb_4-x_Bi_x_S_13_ with different dopant percentage (2-12%).
